# Defective Expression of the Mitochondrial-tRNA Modifying Enzyme GTPBP3 Triggers AMPK-Mediated Adaptive Responses Involving Complex I Assembly Factors, Uncoupling Protein 2, and the Mitochondrial Pyruvate Carrier

**DOI:** 10.1371/journal.pone.0144273

**Published:** 2015-12-07

**Authors:** Ana Martínez-Zamora, Salvador Meseguer, Juan M. Esteve, Magda Villarroya, Carmen Aguado, J. Antonio Enríquez, Erwin Knecht, M.-Eugenia Armengod

**Affiliations:** 1 Laboratory of RNA Modification and Mitochondrial Diseases, Centro de Investigación Príncipe Felipe, Valencia, Spain; 2 Laboratory of Intracellular Protein Degradation and Rare Diseases, Centro de Investigación Príncipe Felipe, Valencia, Spain; 3 Centro de Investigación Biomédica En Red de Enfermedades Raras (CIBERER), node U721, Valencia, Spain; 4 Departamento de Desarrollo y Reparación Cardiovascular, Centro Nacional de Investigaciones Cardiovasculares Carlos III, Madrid, Spain; 5 Departamento de Bioquímica y Biología Molecular y Celular, Facultad de Ciencias, Universidad de Zaragoza, Zaragoza, Spain; Heinrich-Heine-Universität Düsseldorf, GERMANY

## Abstract

GTPBP3 is an evolutionary conserved protein presumably involved in mitochondrial tRNA (mt-tRNA) modification. In humans, *GTPBP3* mutations cause hypertrophic cardiomyopathy with lactic acidosis, and have been associated with a defect in mitochondrial translation, yet the pathomechanism remains unclear. Here we use a *GTPBP3* stable-silencing model (shGTPBP3 cells) for a further characterization of the phenotype conferred by the *GTPBP3* defect. We experimentally show for the first time that GTPBP3 depletion is associated with an mt-tRNA hypomodification status, as mt-tRNAs from shGTPBP3 cells were more sensitive to digestion by angiogenin than tRNAs from control cells. Despite the effect of stable silencing of *GTPBP3* on global mitochondrial translation being rather mild, the steady-state levels and activity of Complex I, and cellular ATP levels were 50% of those found in the controls. Notably, the ATPase activity of Complex V increased by about 40% in GTPBP3 depleted cells suggesting that mitochondria consume ATP to maintain the membrane potential. Moreover, shGTPBP3 cells exhibited enhanced antioxidant capacity and a nearly 2-fold increase in the uncoupling protein UCP2 levels. Our data indicate that stable silencing of *GTPBP3* triggers an AMPK-dependent retrograde signaling pathway that down-regulates the expression of the NDUFAF3 and NDUFAF4 Complex I assembly factors and the mitochondrial pyruvate carrier (MPC), while up-regulating the expression of UCP2. We also found that genes involved in glycolysis and oxidation of fatty acids are up-regulated. These data are compatible with a model in which high UCP2 levels, together with a reduction in pyruvate transport due to the down-regulation of MPC, promote a shift from pyruvate to fatty acid oxidation, and to an uncoupling of glycolysis and oxidative phosphorylation. These metabolic alterations, and the low ATP levels, may negatively affect heart function.

## Introduction

Oxidative phosphorylation (OXPHOS) diseases are a group of multi-systemic and often progressive or fatal disorders that are defined by defects in the OXPHOS system, which affect the cellular ATP supply [1]. The OXPHOS system produces most cellular ATP and consists of ≈85 proteins organized into five multiheteromeric complexes (CI to CV), all of which are immersed in the inner mitochondrial membrane, and two mobile electron shuttles, Coenzyme Q (CoQ) and cytochrome *c*. Complexes CI to CIV (respiratory complexes) are responsible for the oxidation of reducing equivalents (in the form of NADH or FADH_2_) produced by different metabolic pathways, including glycolysis, tricarboxylic acid cycle (TCAC), and oxidation of fatty acids and glutamine. Oxidation of the reducing equivalents is coupled to the pumping of protons (from Complex I, III and IV) into the intermembrane space, and the resulting proton gradient is used by Complex V to synthesize ATP. NADH reducing equivalents are funneled into the mitochondrial electron transport chain through Complex I, whereas FADH_2_ reducing equivalents are incorporated through Complex II or diverse electron transfer flavoproteins (ETFs) such as glycerol-3-phosphate dehydrogenase and ETF-ubiquinone oxidoreductase. Complex II and ETFs transfer electrons to CoQ without creating a transmembrane proton gradient. Respiratory complexes, CoQ, cytochome *c* and ETFs can associate in superstructures with a functional role [2, 3].

Mitochondrial DNA (mtDNA) encodes 13 key OXPHOS proteins (seven of CI, one of CIII, three of CIV, and two of CV) together with the 22 tRNAs and 2 rRNAs required for mitochondrial translation, whereas the nuclear genome encodes the rest of the OXPHOS proteins, as well as more than 30 ancillary factors required for the proper assembly and stability of the OXPHOS complexes [4]. The nuclear genome also provides all the proteins required for the proper functioning of the mitochondrial translation machinery, including proteins responsible for the post-transcriptional modification of mitochondrial tRNAs (mt-tRNAs) and rRNAs [5–7]. Hence, OXPHOS diseases can be due to mutations in either mtDNA or nuclear DNA and a relevant group of these diseases is related to mitochondrial translation defects [5].

Several OXPHOS diseases have been associated with alterations in the post-transcriptional modification of the uridine located at the wobble position of certain mt-tRNAs. They include MELAS (mitochondrial encephalomyopathy and lactic acidosis with stroke-like episodes), MERRF (myoclonic epilepsy and ragged-red fiber), TRMU-dependent acute infantile liver failure and hypertrophic cardiomyopathies dependent on MTO1 and GTPBP3. MELAS and MERRF are due mostly to mutations in the mt-tRNA^Leu(UUR)^ and mt-tRNA^Lys^ genes, respectively [8]. These mutations apparently act as negative identity determinants for the nuclear-encoded enzymes involved in the wobble uridine (U34) modification since mutant tRNAs lack the U34 modifications normally present in their wild-type counterparts [7]. Those enzymes are conserved from bacteria to human. Thus GTPBP3 and MTO1 are the homologs of *Escherichia coli* proteins MnmE and MnmG, respectively, and are thought to be jointly responsible for the synthesis of the taurinomethyl group at position 5 of U34 (τm^5^U) in mt-tRNAs for Leu, Lys, Glu, Gln and Trp, whereas TRMU (also named MTU1) is the homolog of the bacterial MnmA protein and introduces the thiol group at position 2 of U34 (s^2^U) in mt-tRNA^Lys^, mt-tRNA^Glu^, and mt-tRNA^Gln^ [7, 9, 10]. Considering that modifications at U34 optimize the function of mt-tRNAs in mitochondrial translation, it has been proposed that the loss of these modifications in MELAS and MERRF cells is responsible for the onset of the disease [11, 12], although other mechanisms may also be involved [6, 13–16].

*TRMU* (MIM #610230) mutations are associated with reversible infantile respiratory chain deficiency, which is usually accompanied by acute liver failure and, in some cases, with myopathy and neurological symptoms [17–22]. These phenotypes have been ascribed to a mitochondrial translation defect that could be compensated during the first months of life with dietary supplementations [17, 21, 23]. Strikingly, mitochondrial translation in immortalized fibroblasts from one patient and in *TRMU* knocked-down HEK 293 cells has been described to appear normal, despite the drastic drop in the 2-thiouridylation levels of mt-tRNAs [24]. Therefore, the pathomechanism of the *TRMU* mutations remains to be clarified.

Mutations of *MTO1* (MIM #614667) cause infantile hypertrophic cardiomyopathy, lactic acidosis and, in some patients, neurological features [22, 25, 26]. These mutations are usually associated with diminished activities of Complexes I and IV, which could be ascribed to mitochondrial translation impairment after considering the tRNA modifying function of the *MTO1* homologs in yeast and bacteria [9]. However, translation has been reported as being normal in fibroblasts from two affected siblings, despite an approximate 50% reduction in the activity of Complex I or IV [26]. Both patients died early from sudden bradycardia. In contrast, affectation of mitochondrial translation has been recently reported in fibroblasts from one patient who carried a different *MTO1* mutation in homozygosis [27]. The clinical presentation in some *MTO1* patients seemed to depend on the severity of the mutation(s) [25]. However, the disease course was very different for the patients of two families carrying the same mutant genotype, suggesting that protection/risk genetic factors and environmental variations (including pharmacological intervention) may modulate the phenotype [25].

An international collaborative study has recently described the first group of patients (11 individuals from 9 families) with a mitochondrial disorder due to a *GTPBP3* defect [28]. Like *MTO1* mutations, *GTPBP3* mutations (MIM 608536) are mostly associated with hypertrophic cardiomyopathy, lactic acidosis, and combined respiratory chain deficiency. About 50% of identified carriers also exhibited neurological symptoms. One case of atypical presentation with normal respiratory complex activity in muscle was also reported. Analysis of mitochondrial translation in fibroblasts of four affected individuals revealed a severe decrease in three cases, but no detectable effect in a fourth patient who, however, died at 7 months of age from cardiac failure. Therefore, as in the TRMU- and MTO1-dependent diseases, variability in the degree of impairment of the mitochondrial translation has been found among carriers of *GTPBP3* mutations and no unequivocal correlation between the mitochondrial translation defect and the outcome of the disease can be established.

Causes for the inter-individual variability within a specific mt-tRNA modification disease may be, in addition to the mtDNA haplotype, the nuclear background and epigenetic factors, which may modulate the cell response to changes in the functional state of mitochondria. However, this issue has been poorly investigated. Another conundrum with this kind of diseases is how mt-tRNA hypomodification and the subsequent translational stress can cause so different clinical symptoms. These unresolved aspects urge to investigate the signals generated as a result of mt-tRNA hypomodification and the downstream signaling pathways that can modulate the phenotype of the disease.

Here we use a *GTPBP3* stable-silencing cell model (shGTPBP3 cells) to further characterize the phenotype associated with a *GTPBP3* defect. We demonstrate for the first time that the *GTPBP3* defect is associated with an mt-tRNA hypomodification status, as revealed by the greater sensitivity of mt-tRNAs purified from shGTPBP3 cells to digestion by angiogenin. We do not find a consistent general reduction of mitochondrial translation in shGTPBP3 cells, although the ATP content and Complex I activity are reduced to 50% of the control levels. Our data also indicate that stable silencing of *GTPBP3* triggers an AMPK-dependent retrograde signaling pathway that up-regulates the expression of uncoupling protein 2 (UCP2), while down-regulating the expression of both the mitochondrial pyruvate carrier and Complex I assembly factors NDUFAF3 and NDUFAF4. We suggest that altered regulation of fatty acid and glucose metabolism associated with reduced ATP levels contributes to the pathomechanism of the GTPBP3 defect.

## Results

### Down-regulation of *GTPBP3* alters the angiogenin digestion pattern of substrate mt-tRNAs without affecting the 2-thiolation level

**Fig 1:**
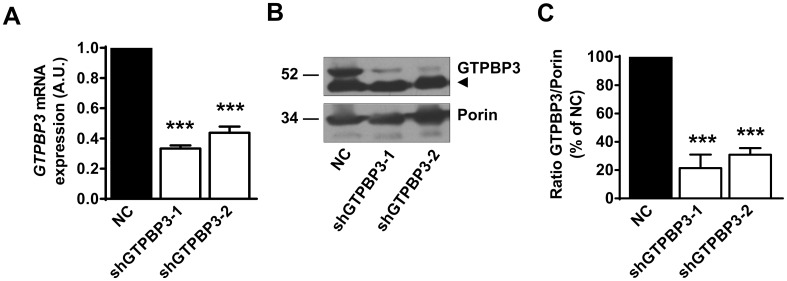
Expression of *GTPBP3* is down regulated in shGTPBP3 cells. **(A)** qRT-PCR analysis of *GTPBP3* mRNA expression in shGTPBP3-1, shGTPBP3-2 and negative control (NC) cells. **(B)** Western blot analysis of GTPBP3 protein in shGTPBP3-1, shGTPBP3-2 and NC cells, using porin as a loading control. Positions of molecular-mass markers (in kDa) are indicated on the left. The arrow denotes a non-specific band. **(C)** Densitometric analysis of GTPBP3 protein normalized to loading control and represented as % of NC. In A and C, mean ± SEM of at least three independent biological replicates. Differences from NC values were found to be statistically significant at ***p<0.001. A.U.: arbitrary units.

To explore the consequences of a *GTPBP3* defect we specifically knocked down the *GTPBP3* expression in HEK-293 cells by RNA interference under stable transfection conditions with short hairpin RNA (shRNA) plasmids. *GTPBP3* silencing in two selected cell lines (shGTPBP3-1 and -2, with each one carrying a different shRNA sequence against *GTPBP3*) was verified at both the RNA and protein levels by quantitative real-time PCR and Western blotting, respectively (Fig 1). The steady-state levels of the GTPBP3 protein in shGTPBP3 cells decreased to about 75% compared with those of the negative control (NC). This decrease was somewhat larger than that found in mRNA levels (about 60%). The difference could probably be due to the activity of a translational regulatory mechanism that is affected in shGTPBP3 cells. However, its specific nature is at present unknown since the available information on the regulation of GTPBP3 expression is scarce [16].

**Fig 2:**
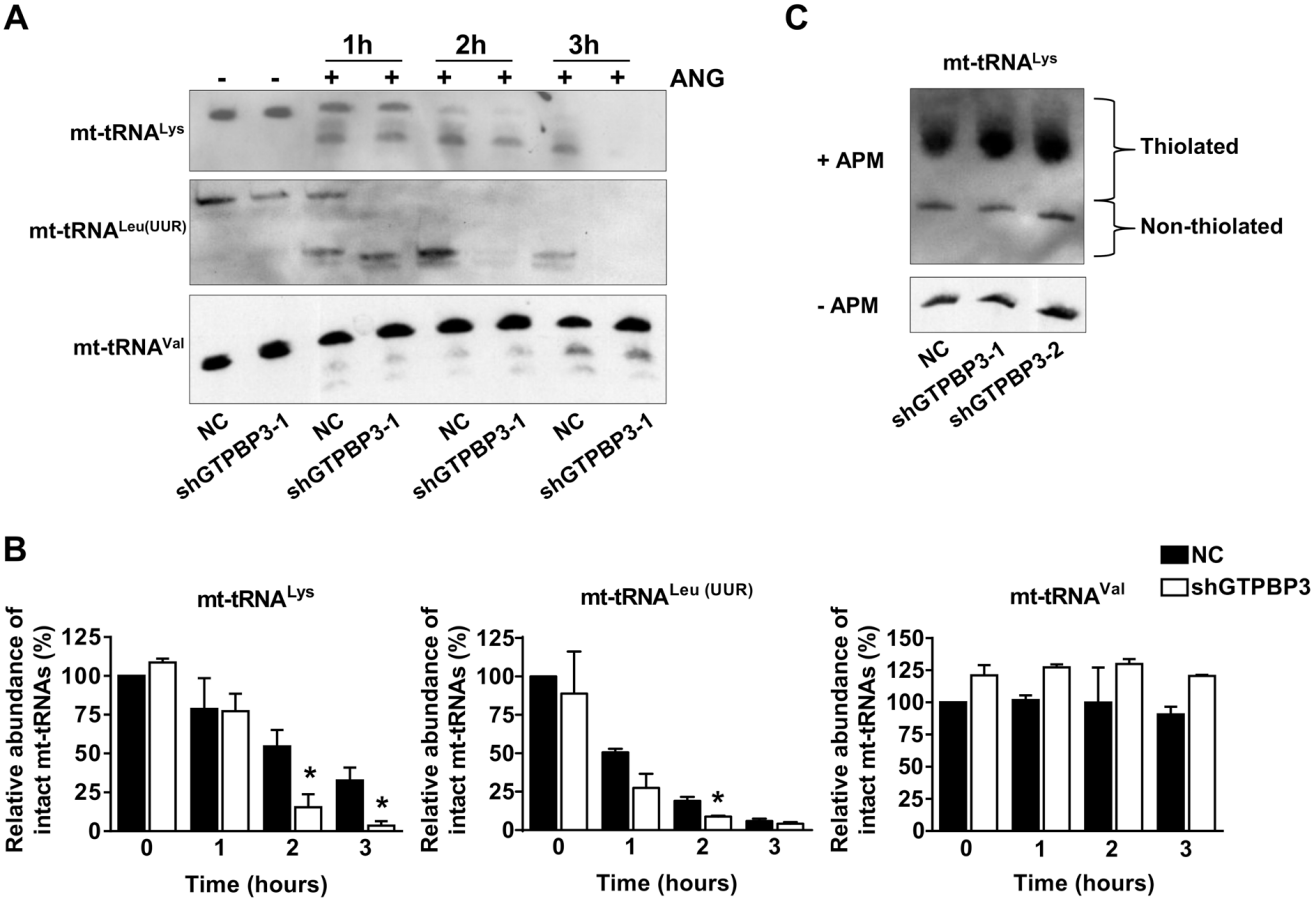
Altered angiogenin digestion pattern of mt-tRNA^Lys^ and mt-tRNA^Leu(UUR)^ purified from shGTPBP3 cells. **(A)** Northern analysis of mt-tRNA^Lys^ (upper panel), mt-tRNA^Leu(UUR)^ (middle panel) and mt-tRNA^Val^ (lower panel) molecules after *in vitro* angiogenin (ANG) digestion of small RNAs purified from shGTPBP3-1 and negative control (NC) cells for 1, 2 and 3 h. Similar results were obtained with shGTPBP3-2. **(B)** Relative abundance of intact mt-tRNAs after angiogenin treatment. The amount of intact mt-tRNA after 0, 1, 2 and 3 h of incubation with angiogenin was quantified from blots similar to those shown in panel A and is represented as percentage of the undigested NC sample (0 h). Data are the mean ± SEM of at least three independent biological replicates. Differences were found to be statistically significant at *p<0.05. **(C)** APM-Northern analysis of the 2-thiolation status of mt-tRNA^Lys^ molecules from NC, shGTPBP3-1 and shGTPBP3-2 cells. The same amount of total RNA (7.5 μg) was run in a denaturing polyacrylamide-urea gel in the presence (+) or absence (-) of APM. The thiolated tRNAs were detected as retarded bands in the presence of APM. Experiments were performed with RNAs purified from at least three independent cultures of each cell line, with similar results to those shown in the panel.

Next, we decided to study the effect of the GTPBP3 depletion on mt-tRNA modification. Unfortunately, a direct analysis of the τm^5^ modification of U34 is a very difficult task because of the low abundance of mt-tRNAs relative to their cytosolic homologues, which limits their availability from biological samples [7, 29]. In fact, the taurine modification deficiency has not been evaluated in carriers of *GTPBP3* mutations [28]. We thus addressed this issue by analyzing the sensitivity of substrate and non-substrate mt-tRNAs of GTPBP3 to digestion with angiogenin, a tRNA-specific enzyme of the RNase A superfamily [30–35]. This approach was based on previous findings indicating that loss of certain tRNA modifications increases the angiogenin-mediated cleavage of cytosolic tRNAs in *Drosophila*, mouse and human [30, 34]. After *in vitro* angiogenin digestion of small RNA obtained from shGTPBP3-1 and NC cells, followed by Northern blot analysis (Fig 2A), we found that the GTPBP3-substrates mt-tRNA^Lys^ and mt-tRNA^Leu(UUR)^ were more sensitive to angiogenin when purified from shGTPBP3 cells than when obtained from the control cells (Fig 2A and 2B). In contrast, we found no differences in the digestion patterns of a non-substrate tRNA of GTPBP3 (mt-tRNA^Val^). These results suggest that the higher susceptibility towards cleavage by angiogenin of mt-tRNA^Lys^ and mt-tRNA^Leu(UUR)^ obtained from stable *GTPBP3* knocked-down cells is due to the deficit of the τm^5^ group.

Notably, mt-tRNA^Lys^ purified from shGTPBP3 cells showed normal 2-thiolation levels at U34, according to the migration pattern observed on [(*N*-acryloylamino)phenyl]mercuric chloride (APM)-Northern blots (Fig 2C), in which the APM polymerized in the gel causes a specific retardation of thio-modified tRNAs through its binding to the sulfur in the tRNA. The modification status of mt-tRNA^Lys^ in shGTPBP3 cells suggests that thiolation at position 2 of mt-tRNA^Lys^ occurs independently of the presence of a modification at position 5, similarly to that observed in *E*. *coli* tRNAs and *S*. *cerevisiae* mt-tRNAs [10, 36].

To verify the suitability of the angiogenin-based approach, we used native tRNA^Lys^_mnm5s2UUU_ purified from *E*. *coli* strains as a substrate for the enzyme since, in this case, it is possible to compare the results of the angiogenin assay with those of a direct analysis of the tRNA nucleoside composition by HPLC after digesting tRNA with nuclease P1 and bacterial alkaline phosphatase [37]. We found that digestion of the *E*. *coli* tRNA^Lys^_mnm5s2UUU_ purified from a null *mnmE* mutant strain (which lacks the MnmE protein; *i*.*e*., the GTPBP3 homologue) was more sensitive to angiogenin cleavage than when obtained from a wild-type *E*. *coli* strain (S1A Fig). These data perfectly fit with the presence (in the wild-type strain) or the absence (in the *mnmE* mutant strain) of the MnmE-dependent modification at position 5 of U34, as determined by HPLC analysis of tRNA^Lys^_mnm5s2UUU_ hydrolysates (S1B Fig). It should be noted that a 2-thiolated U34 was present in the tRNA purified from the *mnmE* mutant, indicating that modification at position 2 is independent of modification at position 5. The results obtained with the bacterial system support the notion that sensitivity towards angiogenin depends on the MnmE-mediated modification. It is then reasonable to assume that the greater angiogenin sensitivity of mt-tRNA^Lys^ and mt-tRNA^Leu(UUR)^ purified from shGTPBP3 cells is due to the absence of the GTPBP3-dependent modification. Therefore, the angiogenin-based experimental approach used herein appears as an effective and affordable strategy to evaluate the modification status of U34 at position 5. Thus our data offer the first experimental evidence for a role of human GTPBP3 in modification of mt-tRNAs.

### Characterization of mitochondrial dysfunction in shGTPBP3 cells

**Fig 3:**
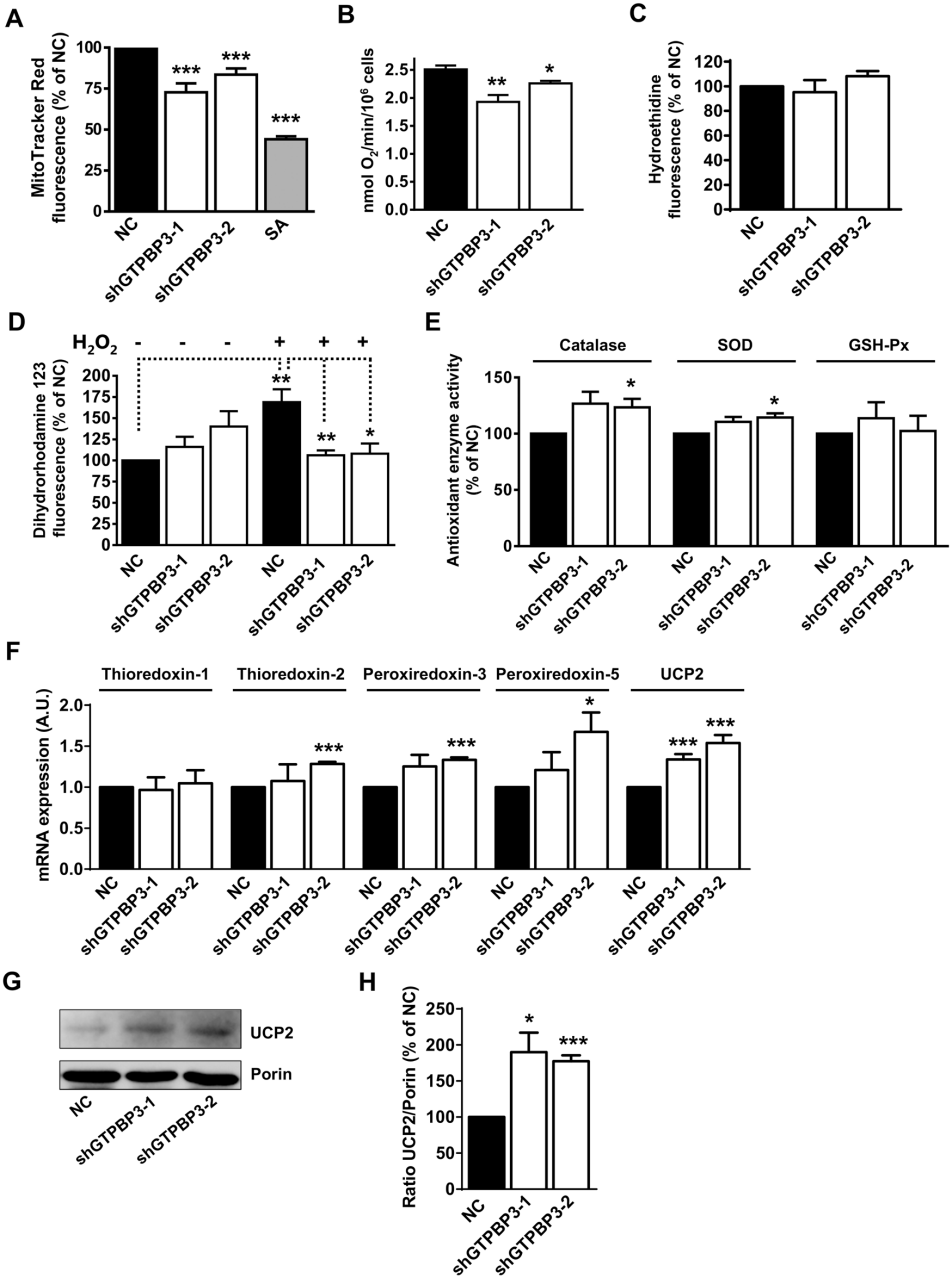
shGTPBP3 cells show increased antioxidant capacity. **(A)** Determination of the mitochondrial membrane potential by flow cytometry in shGTPBP3-1, shGTPBP3-2 and negative control (NC) cells with the fluorescent probe MitoTracker Red. NC cells treated for 30 min with sodium azide (SA) at 25 mM were included in the analysis as a positive control for the membrane potential drop. **(B)** Determination of oxygen consumption rate with a Clark-type oxygen electrode in shGTPBP3-1, shGTPBP3-2 and NC cells. **(C)** Determination of ROS by flow cytometry in shGTPBP3-1, shGTPBP3-2 and NC cells with hydroethidine. **(D)** Determination of ROS by flow cytometry in shGTPBP3-1, shGTPBP3-2 and NC cells treated (+) or not (-) for 2 h with 0.3 mM H_2_O_2_ with dihydrorhodamine 123. **(E)** Measurements of antioxidant enzyme activities: Catalase, SOD (superoxide dismutase) and GSH-Px (glutathione peroxidase). Data in A, C, D and E are expressed as % of NC. **(F)** qRT-PCR analysis of *Thioredoxin-1*, *Thioredoxin-2*, *Peroxiredoxin-3*, *Peroxiredoxin-5* and *Uncoupling protein-2* (*UCP2*) mRNA expression in shGTPBP3-1, shGTPBP3-2 and NC cells. **(G)** Western blot analysis of UCP2 in shGTPBP3-1, shGTPBP3-2 and NC cells. The filter was also probed with porin as a loading control. **(H)** Densitometric analysis of UCP2 normalized to loading control and represented as % of NC. All data are the mean ± SEM of at least three independent biological replicates. Differences from NC values were found to be statistically significant at *p<0.05, **p<0.01 and ***p<0.001. A.U.: arbitrary units.

To assess the effect of *GTPBP3* down-regulation on mitochondrial physiology, we firstly evaluated the mitochondrial membrane potential and oxygen consumption by MitoTracker Red tracked by flow cytometry and polarography with a Clark-type electrode, respectively. shGTPBP3 cells exhibited a decrease in both parameters (Fig 3A and 3B).

Next we evaluated the oxidative stress in shGTPBP3 cells and found that the levels of two different ROS markers did not significantly change compared to NC cells (Fig 3C and 3D). These data differed from those previously reported after transiently knocking down *GTPBP3* in the same cell line, where an important increase (28%) in superoxide anion levels was observed [38]. This finding suggests that in a long-term process like the stable knockdown expression of *GTPBP3*, the cell has time to mount an adaptive response, which results in an increased antioxidant capacity. In agreement with this hypothesis, exposure to 0.3 mM H_2_O_2_ for 2 h resulted in increased intracellular levels of H_2_O_2_ in NC cells, but not in shGTPBP3 cells (Fig 3D).

We then looked at the activity or expression of antioxidant systems. No consistent increase in the activity of catalase, superoxide dismutase and glutathione peroxidase was observed in shGTPBP3 cells (Fig 3E). We analyzed the transcription levels of some components of the thioredoxin system, a major antioxidant system that maintains redox balance through the action of thioredoxin and thioredoxin reductase, and regulates the activity of peroxiredoxins [39, 40]. We observed a tendency towards an increased expression of thioredoxin-2, peroxiredoxin-3 and peroxiredoxin-5 (Fig 3F), all of them with a mitochondrial localization [39, 41]. Most important was the increase in both mRNA and protein levels of UCP2 (Fig 3F–3H), whose regulation is known to occur at several levels. Uncoupling protein 2 (UCP2) has been associated with protective functions against excessive ROS production, but its precise function is still a matter of debate [42–49]. Several recent reports support the view that UCP2 promotes a metabolic shift from pyruvate to glutamine and fatty acid oxidation as a means to provide FADH_2_ reducing equivalents to the respiratory chain [42, 44, 46, 47, 49], thus lowering ROS production as a secondary effect of its metabolic control rather than by directly uncoupling mitochondrial respiration from ATP synthesis. Whatever the precise function of UCP2 is, the nearly 2-fold increase of this protein in shGTPBP3 cells suggests that it contributes to the increased antioxidant capacity of these cells.

**Fig 4:**
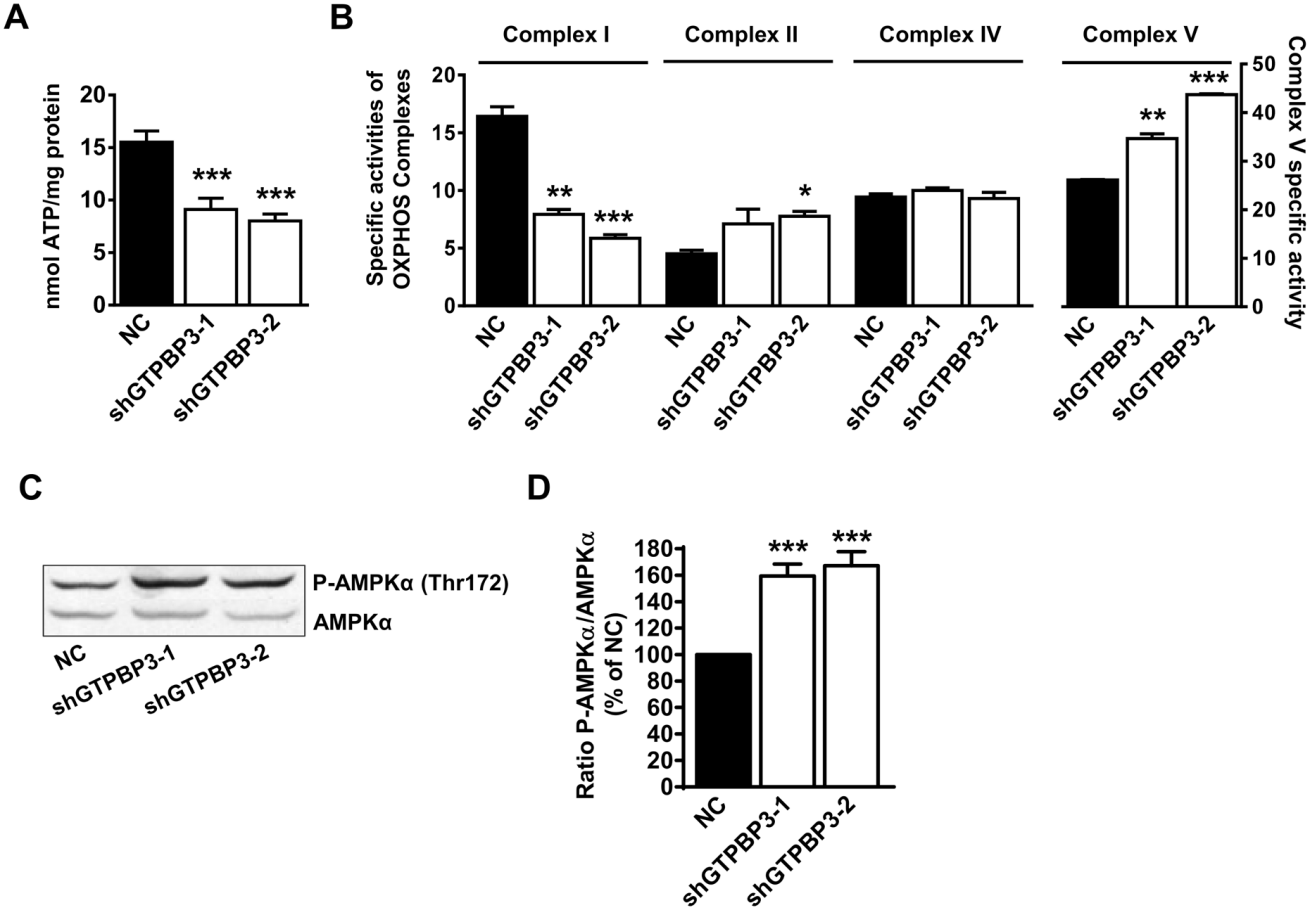
ATP levels and Complex I activity are reduced in shGTPBP3 cells whereas AMPK is activated. **(A)** ATP levels in shGTPBP3-1, shGTPBP3-2 and negative control (NC) cells. **(B)** Measurement of mitochondrial Complex I, II, IV and V activities. All activities are expressed as citrate synthase ratios ((nmol/min/mg protein)/(citrate synthase-specific activity) x 100). **(C)** Western blot analysis of phospho-Thr172-AMPKα in shGTPBP3-1, shGTPBP3-2 and NC cells. The filter was also probed with AMPKα as a loading control. **(D)** Densitometric analysis of phospho-Thr172-AMPKα normalized to the loading control and represented as % of NC. All data are the mean ± SEM of at least three independent biological replicates. Differences from NC values were found to be statistically significant at *p<0.05, **p<0.01 and ***p<0.001.

Interestingly, the ATP levels in shGTPBP3 cells were notably lowered (≈50%) compared to the control cells (Fig 4A), which suggests an impairment of oxidative phosphorylation. Indeed, the activity of Complex I, but not of Complex IV, was found to be drastically reduced in GTPBP3 depleted cells (Fig 4B). We also observed a tendency towards an increase in Complex II activity and a significant increase in ATPase activity of Complex V (Fig 4B), which could compensate for some effects of Complex I deficiency. On the one hand, an increase in Complex II activity might boost the delivery of reducing equivalents to the OXPHOS system from succinate. On the other hand, a reverse operation of Complex V could help to prevent a drastic drop of the membrane potential in shGTPBP3 cells. In this respect, it has been previously shown that when Complex I is inhibited, the membrane potential may be maintained by the reversal of the F_0_F_1_-ATPase, although the mechanisms by which F_0_F_1_-ATPase operates and reverses are not fully understood [50].

One key cellular sensor of the metabolic status is the AMP-activated protein kinase (AMPK), which is activated by a number of mechanisms when the AMP:ATP ratio increases [51–54]. Since ATP levels are reduced in shGTPBP3 cells, we evaluated AMPK activation by Western blot analysis and found that phosphorylation of AMPKα at Thr172 was indeed stimulated in these cells (Fig 4C and 4D).

**Fig 5:**
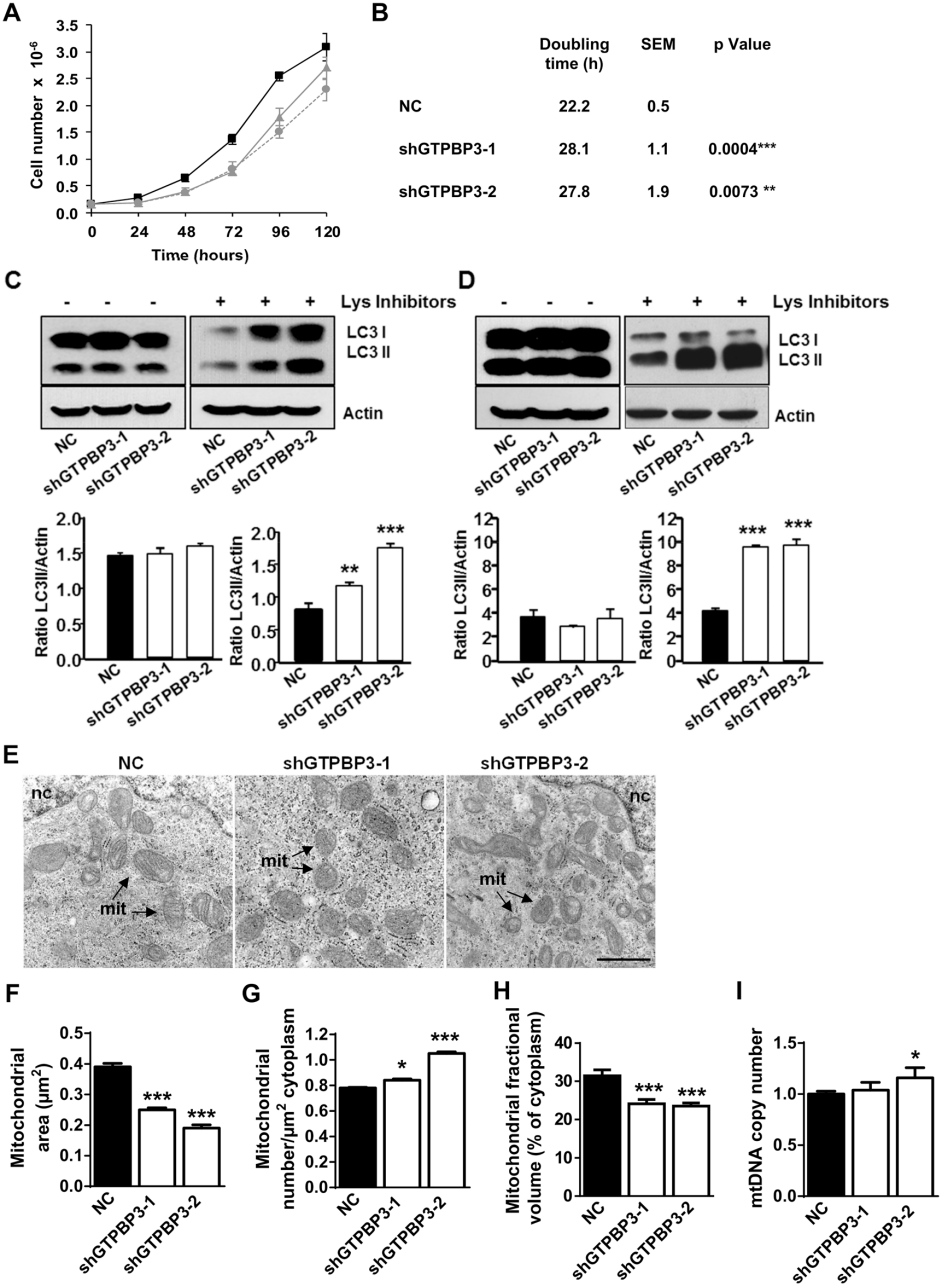
Stable knocking-down of *GTPBP3* expression alters cell growth, autophagy, and the mitochondrial size and fractional volume, yet the relative mtDNA copy number is maintained. **(A)** Growth curves of negative control (NC) (black square), shGTPBP3-1 (grey triangle) and shGTPBP3-2 (grey circle) cells. **(B)** Doubling time (h) of shGTPBP3-1, shGTPBP3-2 and NC cells. **(C, D)** Representative immunoblots, using antibodies which recognize LC3 or, as a loading control, actin, with extracts of shGTPBP3-1, shGTPBP3-2 and NC cells. The cells were incubated for 1 h with (+) or without (-) lysosomal (Lys) inhibitors and under conditions of low (full medium, **C**) or high (phosphate buffered saline, **D**) proteolysis. The positions of LC3-I and LC3-II bands are indicated on the left. The histograms below show the densitometric measurements of LC3-II normalized to loading control and represented as % of NC from three independent experiments. **(E)** Representative electron micrographs of NC, shGTPBP3-1 and shGTPBP3-2 cells (left, middle and right panel, respectively). Nuclei (nc) and some mitochondria (mit) are indicated. Bar: 1 μm. **(F-H)** Mean mitochondrial area (F), mitochondrial number per μm^2^ of cytoplasm (G) and mitochondrial fractional volume expressed as μm^3^ mitochondria/100 μm^3^ cytoplasm (H) in shGTPBP3-1, shGTPBP3-2 and NC cells. **(I)** Quantification of the mitochondrial-encoded *COX2* gene relative to the nuclear-encoded *SDH* gene in shGTPBP3-1, shGTPBP3-2 and NC cells by qPCR. All data are the mean ± SEM of at least three independent biological replicates. Differences from NC values were found to be statistically significant at *p<0.05 and ***p<0.001.

Mitochondria are intimately involved in cell signaling pathways, and thus participate in the regulation of key processes, including cell growth and macroautophagy, hereafter simply called autophagy [53, 55, 56]. OXPHOS dysfunction has been shown to affect these processes to some extent [57–61]. In this respect, we found that cell growth was reduced in shGTPBP3 cells (Fig 5A and 5B), while autophagic activity, measured both under low (Fig 5C) and high (Fig 5D) proteolysis conditions and in the presence of lysosomal inhibitors, appeared to be increased, as we observed accumulation of LC3-II (microtubule-associated light-chain 3), a hallmark of autophagy.

Interestingly, mitochondria were smaller and somewhat more abundant in shGTPBP3 cells (Fig 5E–5G). Mitochondrial fragmentation, due to a fission-fusion imbalance, is a common stress response that is required principally to segregate and eliminate dysfunctional mitochondria via a process of selective autophagy called mitophagy [55, 62–64]. Depletion of ATP is known to trigger general fragmentation of the mitochondrial web due to cleavage of Opa1, a protein involved in mitochondrial fusion [62]. Notably, the mitochondrial fractional volume was found to be reduced in shGTPBP3 cells (Fig 5H), which could be related to increased autophagy aimed at removing damaged mitochondria. It is noteworthy that the mtDNA copy number did not lower (Fig 5I), a feature which, in this context, could be associated with the activation of an mtDNA maintenance program given that the mtDNA copy number can be modulated according to the energy requirements of the cell [65]. It is known that AMPK activation can enhance mtDNA biogenesis, promoting at the same time the clearance of defective mitochondria while suppressing cell growth [52, 64, 66]. Therefore, activation of AMPK might contribute to the slower growth, the increased autophagy and the maintenance of the relative mtDNA copy number exhibited by shGTPBP3 cells.

### GTPBP3 knockdown causes a mild deficiency in mitochondrial translation

**Fig 6:**
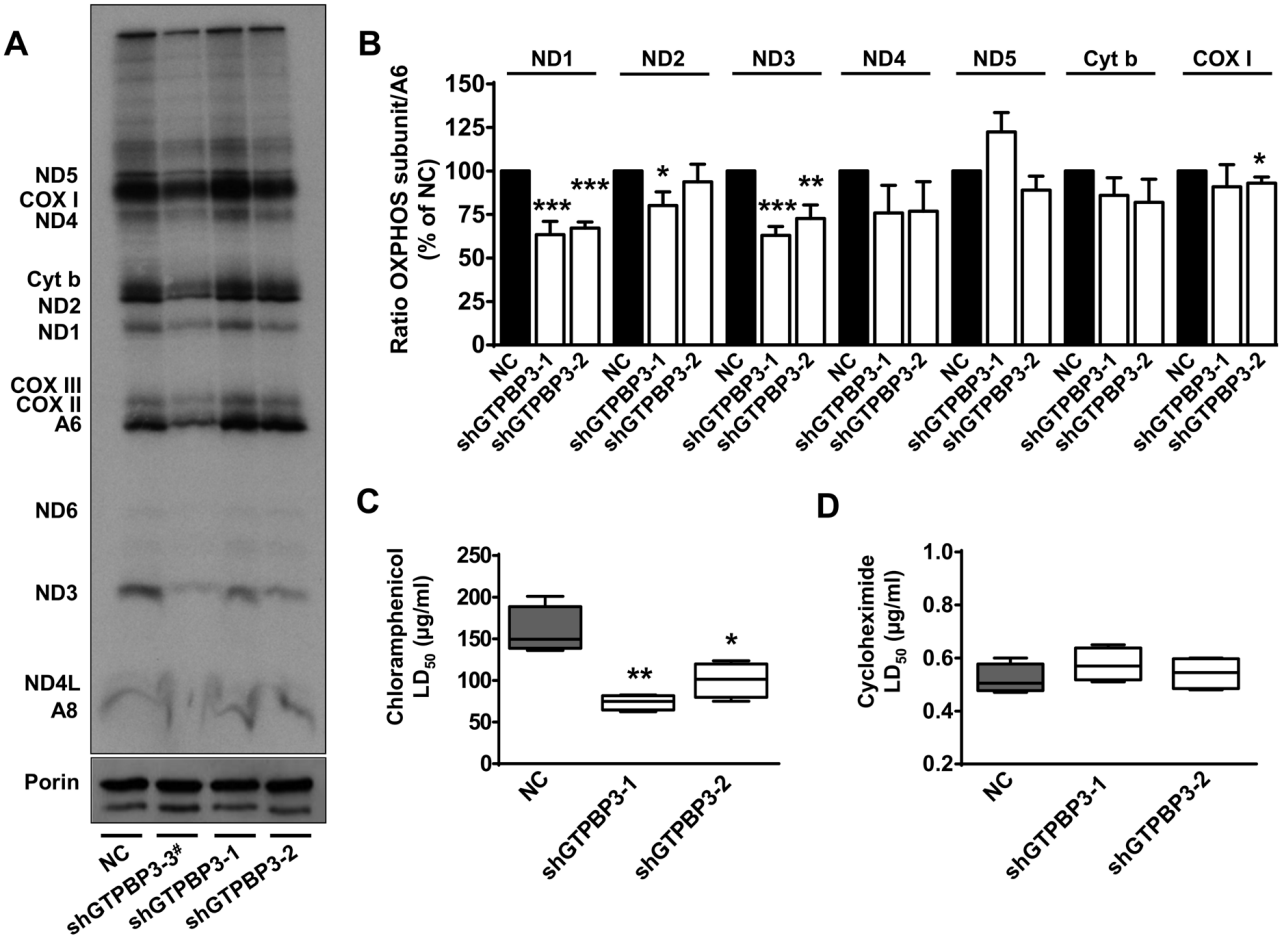
Mitochondrial translation is mildly affected by GTPBP3 knockdown. **(A)** Analysis of *de novo* mitochondrial translation in shGTPBP3-1, shGTPBP3-2 and negative control (NC) cells, pulse-labeled with [^35^S]-methionine for 1 h in the presence of emetine (ND1 to ND6: NADH dehydrogenase subunits 1 to 6; CYTB: Cytochrome b; COXI, II and III: Cytochrome C oxidase subunits I to III; A6 and A8: ATP synthase subunits 6 and 8). ^#^shGTPBP3-3 cell line was excluded from further analysis due to instable down-regulation. **(B)** Densitometric analysis of radiolabeled OXPHOS subunits normalized to subunit A6 of Complex V and represented as % of NC. Data are mean ± SEM of three independent biological replicates. **(C and D)** Differential influence of chloramphenicol (D) and cycloheximide (E) on the viability of NC versus shGTPBP3-1 and shGTPBP3-2 cells. Boxes represent the interquartile range. The middle line represents the median and the whisker-box plots represent minimum and maximum observations *p<0.05, **p<0.01 and ***p<0.001.

To study the effect of stable knock down of *GTPBP3* on mitochondrial translation, shGTPBP3 and NC cells were pulse-labeled with a mixture of [^35^S]-methionine and [^35^S]-cysteine in the presence of emetine, a cytoplasmic translation inhibitor, and mitochondrial translation products were analyzed by SDS-PAGE. No changes were observed in shGTPBP3 cells in either the overall protein synthesis or migration of any polypeptide, although in both shGTPBP3 clones, specific decreases in labeling of the Complex I ND1 and ND3 subunits in relation to either subunit A6 (Complex V) or cytochrome b (Complex III) were significant (Fig 6A and 6B; data not shown).

Puzzling observations for the effect of mutations in genes encoding components of the mitochondrial translation apparatus have been frequently reported [18, 23, 24, 26–28, 67]. This suggests that either a drop in mitochondrial translation should be severe if it is to be revealed by certain methodologies, or compensatory mechanisms, whose expression may depend on the genetic background and cell type, help improve mitochondrial translation efficiency. Therefore, in order to get a more sensitive approach, we decided to treat cells with chloramphenicol, a mitochondrial translation inhibitor, reasoning that if mitochondrial protein synthesis was affected to some extent in shGTPBP3 cells, they could be more sensitive to the drug. We found that the LD_50_ for chloramphenicol was significantly lower for these cells than for the control cells, whereas the LD_50_ for cicloheximide, a specific inhibitor of the cytoplasmic ribosomes, was similar for both cell types (Fig 6C and 6D). These results suggest that mitochondrial translation stress exists in shGTPBP3 cells, which is increased or synergized by the binding of chloramphenicol to the A site of the mitoribosome.

### Impairment of *GTPBP3* expression causes down-regulation of Complex-I assembly factors NDUFAF3 and NDUFAF4

**Fig 7:**
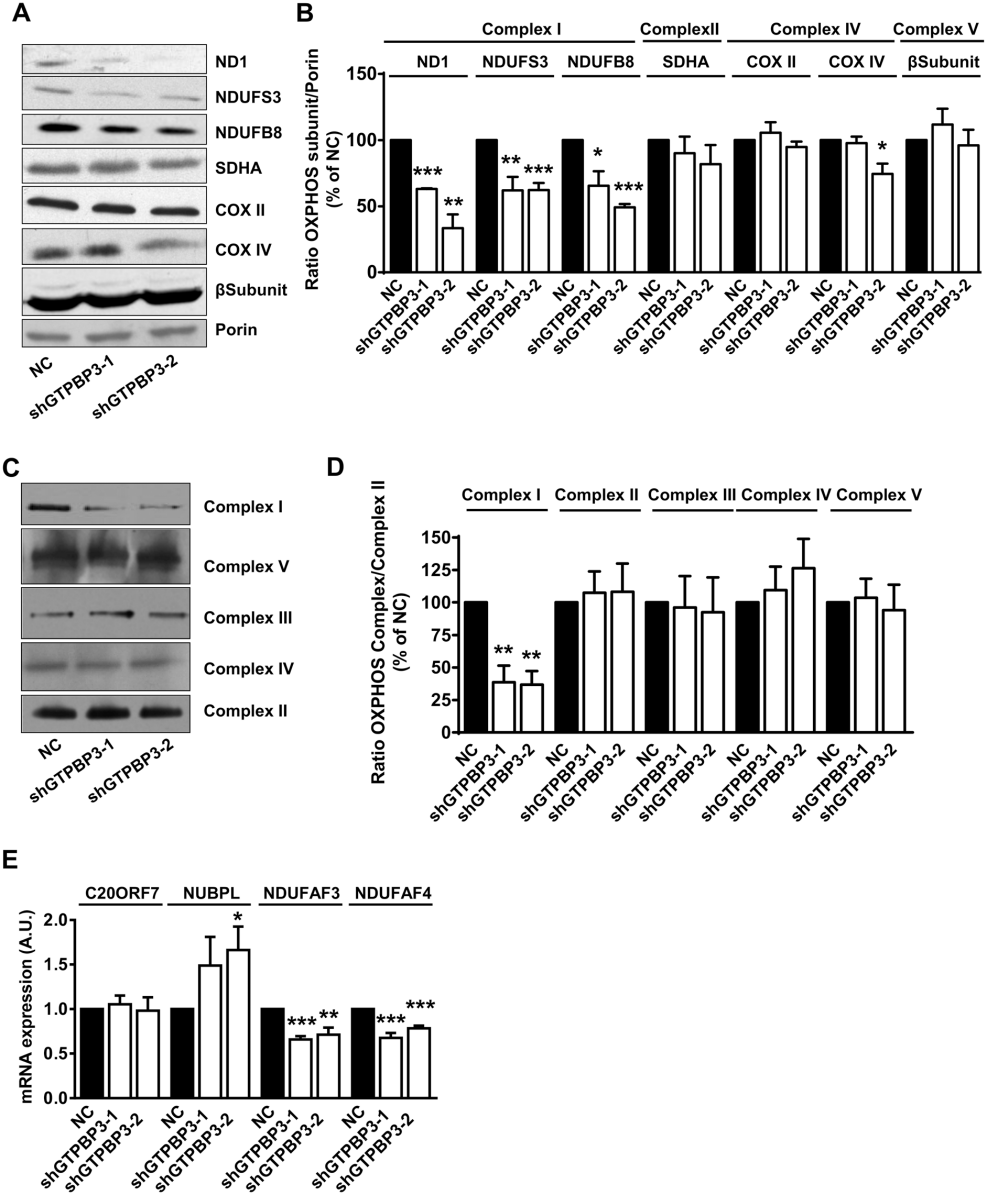
**Stable knock-down of GTPBP3 disturbs Complex I assembly and reduces the expression of Complex I assembly factors NDUFAF3 and NDUFAF4 (A)** Western blot analysis of OXPHOS subunits ND1, NDUFS3 and NDUFB8 (Complex I), SDHA (Complex II), COXII and COXIV (Complex IV), and β-subunit (Complex V) in shGTPBP3-1, shGTPBP3-2 and negative control (NC) cells. The filter was also probed with porin as a loading control. **(B)** Densitometric analysis of OXPHOS subunits normalized to porin and represented as % of NC. **(C)** Representative Blue Native-PAGE of OXPHOS complexes in shGTPBP3-1, shGTPBP3-2 and NC cells. **(D)** Densitometric analysis of OXPHOS Complexes normalized to Complex-II (loading control) and represented as % of NC. **(E)** qRT-PCR analysis of *C20ORF7*, *NUBPL*, *NDUFAF3* and *NDUFAF4* mRNA expression in shGTPBP3 and NC cells. All data are the mean ± SEM of at least three independent biological replicates. Differences from NC values were found to be statistically significant at *p<0.05, **p<0.01 and ***p<0.001. A.U.: arbitrary units.

One possible explanation for the reduced activity of Complex I in shGTPBP3 cells, despite the relatively unaffected levels of *de novo* synthesis of mitochondrial proteins, may be that post-translational processing is altered. Therefore, we analyzed the steady-state levels of some nuclear- and mitochondrial-encoded subunits of Complexes I and IV, the main affected complexes in patients carrying *GTPBP3* mutations [28], and also of some nuclear-encoded subunits of Complex II and V (Fig 7A and 7B). We observed a decrease in the levels of proteins from Complex I (the mtDNA-encoded ND1 protein and the nuclear-encoded NDUFS3 and NDUFB8 proteins) in both shGTPBP3 cell lines, and a decrease in the nuclear-encoded subunit of Complex IV COX IV in only one shGTPBP3 cell line (shGTPBP3-2). No significant effect on the steady-state levels was detected for Complex-II SDHA subunit, Complex-IV COX II subunit, and Complex-V β-subunit. It is stressed that the decrease of proteins NDUFS3 and NDUFB8 was not consistently accompanied by a similar decline in the respective mRNA levels (S2 Fig), which suggests that translation and/or stability of both proteins is altered in shGTPBP3 cells.

Then we assessed the steady-state levels of fully assembled complexes by blue native polyacrylamide gel electrophoresis (BN-PAGE) analysis followed by Western blotting. The analysis revealed a marked decrease in the Complex I levels in shGTPBP3 cells, whereas the other complexes remained unchanged (Fig 7C and 7D). Longer exposure of blots did not reveal any accumulation of Complex I assembly intermediates (data not shown), suggesting that the impairment of Complex I assembly occurs early in shGTPBP3 cells.

The ND1 subunit participates in the early formation of a ∼400 kDa subcomplex which nucleates the assembly process of Complex I [4, 68]. Therefore, a defect in ND1 synthesis could be responsible for the small amounts of this complex in shGTPBP3 cells. Notwithstanding, the effect of the stable knocking-down of *GTPBP3* expression on the steady-state levels of the ND1 subunit and Complex I (Fig 7B and 7D) appeared to be somewhat more severe than the effect on ND1 synthesis (Fig 6B). This hints at a perturbation of the stability/assembly of ND1 and Complex I that could be independent of ND1 translation. It has been shown that siRNA-mediated knock-down of early Complex I assembly factors abrogated the assembly of this complex, and resulted in the rapid proteolysis of newly synthesized ND1 [69]. Considering these data, we decided to analyze the expression of such assembly factors and found that the *NDUFAF3* and *NDUFAF4* mRNA levels were significantly reduced in shGTPBP3 cells (Fig 7E).

It is noteworthy that in mitochondria derived from patients carrying mutations in the *NDUFAF3* gene, Complex I assembly was disrupted without accumulation of assembly intermediates [70], which is reminiscent of what we observed in shGTPBP3 cells. Proteins NDUFAF3 and NDUFAF4 are codependent as knocking down the expression of one of them leads to a simultaneous decrease in the level of both proteins [70]. This suggests that the decrease in *NDUFAF3* and *NDUFAF4* mRNA expression found in shGTPBP3 cells (Fig 7E) may have a significant impact on the protein levels of both factors and, consequently, on Complex I assembly. Therefore, we propose that the stable knocking down of *GTPBP3* triggers a retrograde signaling pathway that down-regulates the expression of *NDUFAF3* and *NDUFAF4* and this, in turn, affects Complex I assembly and contributes to a rapid turnover of the ND1 subunit. According to this proposal, the ND1 decrease observed in the pulse-labeling experiments (Fig 6B) may be due, at least in part, to rapid proteolysis of the newly synthesized protein.

### AMPK signaling results in down-regulation of Complex I assembly factors and mitochondrial pyruvate carrier, while up-regulating UCP2 expression

**Fig 8:**
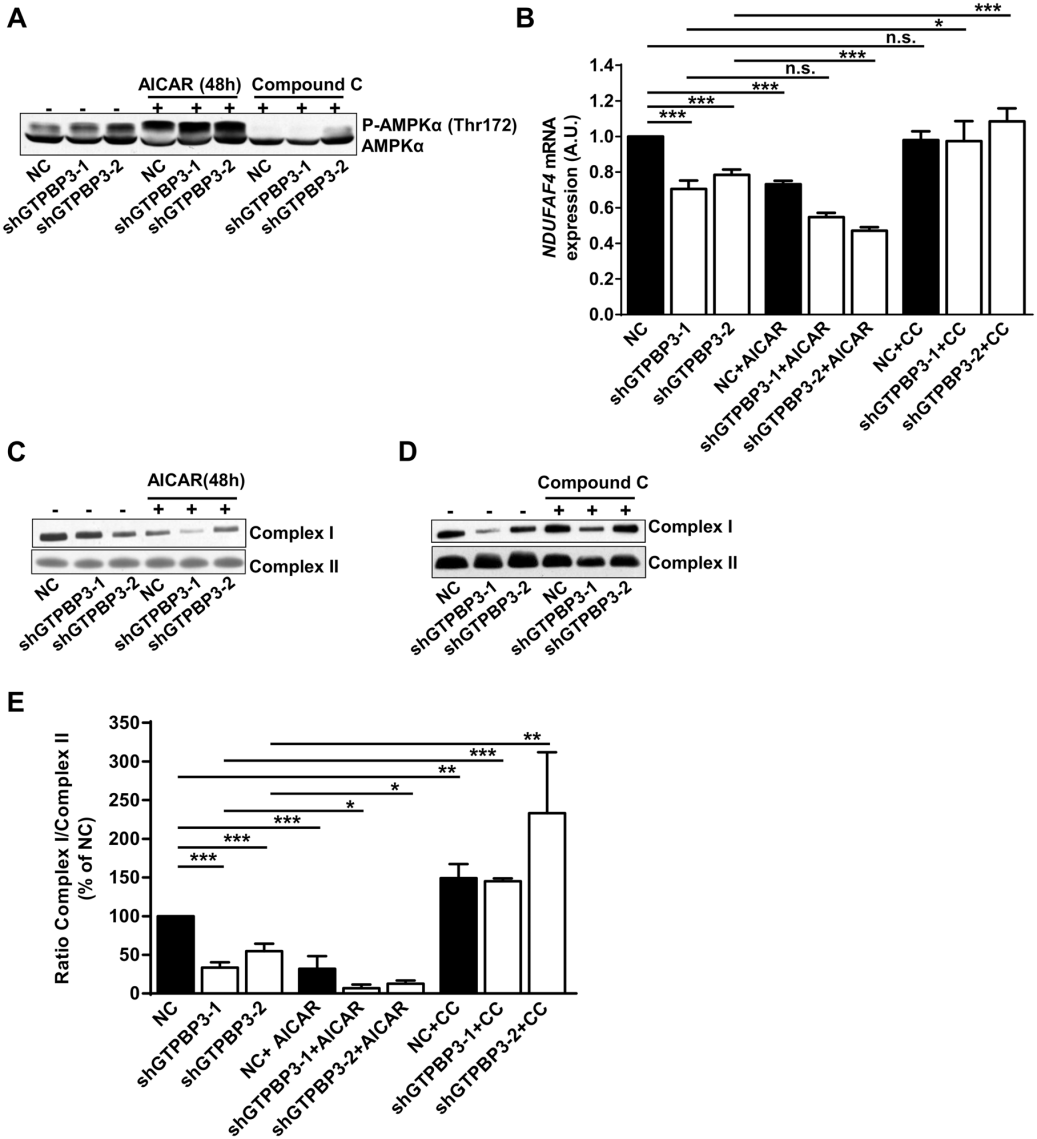
AMPK contributes to the down regulation of NDUFAF4. **(A)** Western blot analysis of phospho-Thr172-AMPKα in shGTPBP3-1, shGTPBP3-2 and negative control (NC) cells treated (+) or not (-) with AMPKα activator AICAR (1 mM) for 48 h or with AMPKα inhibitor Compound C (5 μM) for 1 h. The filter was also probed with AMPKα. **(B)** qRT-PCR analysis of *NDUFAF4* mRNA expression in shGTPBP3-1, shGTPBP3-2 and NC cells treated or not with AICAR or with Compound C (CC) as in (A). **(C and D)** Blue Native-PAGE analysis of Complex I in shGTPBP3-1, shGTPBP3-2 and NC cells treated (+) or not (-) with AICAR (C) or with Compound C (D) as in (A). **(E)** Densitometric analysis of Complex I normalized to the loading control and represented as % of NC. All data are the mean ± SEM of at least three independent biological replicates. Differences were found to be statistically significant at *p<0.05, **p<0.01, ***p<0.001. n.s.: non-significant differences. A.U.: arbitrary units.

AMPK regulates almost all aspects of cellular function and mediates adaptive responses of cells with mitochondrial dysfunction [51, 52, 54, 61]. Therefore, we wondered whether the observed down-regulation of NDUFAF3 and NDUFAF4 in shGTPBP3 cells could be related to AMPK activation. To address this question, we first treated shGTPBP3 and NC cells for 48 h with 1 mM 5-aminoimidazole-4-carboxamide-1-*β*-D-ribofuranoside (AICAR), a widely used AMPK activator. This treatment led to a significantly increased AMPK phosphorylation in all cases (Fig 8A), and to a concomitant reduction of the NDUFAF4 mRNA levels (Fig 8B) in NC cells and also, but to a lesser extent, in shGTPBP3 cells. When cells were treated for 1 h with 5 μM of the AMPK inhibitor compound C, which strongly inhibited AMPK phosphorylation in both NC and shGTPBP3 cells (Fig 8A), the NDUFAF4 mRNA levels in shGTPBP3 cells increased to reach the values of NC cells (Fig 8B). Altogether, these results suggest that AMPK activation mediates down-regulation of the *NDUFAF4* gene.

A decrease in the amount of the assembly factor NDUFAF4 is expected to lower the levels of NDUFAF3 (and vice versa), and to affect biogenesis of Complex I [69, 70]. In agreement with this notion, we found that the steady-state levels of Complex I lowered after treating NC and shGTPBP3 cells with AICAR (Fig 8C and 8E). In contrast, treatment of cells with the AMPK inhibitor compound C led to the stabilization, and therefore, accumulation of Complex I (Fig 8D and 8E).

**Fig 9:**
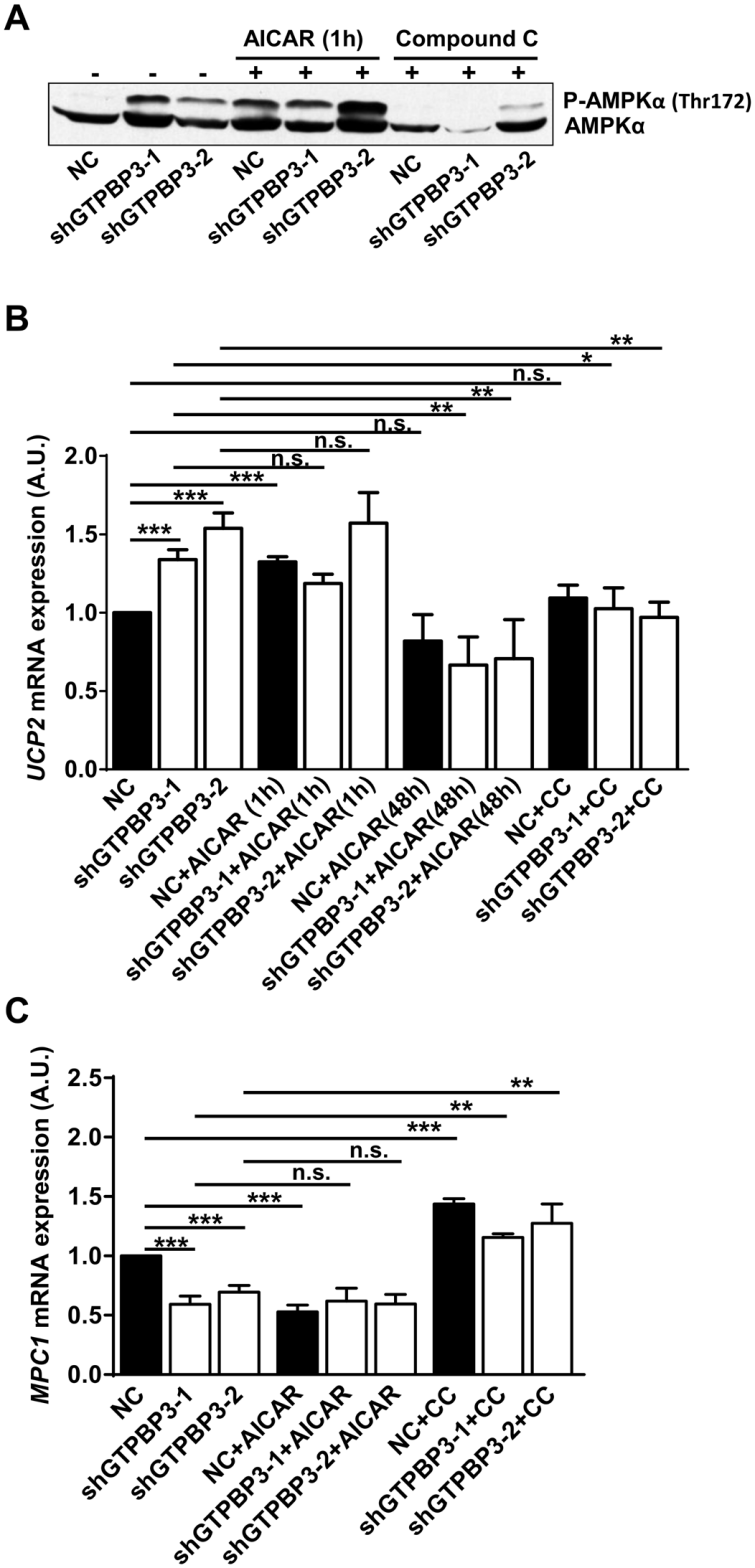
AMPK activation contributes to the induction of UCP2 and the down-regulation of MPC1. **(A)** Western blot analysis of phospho-Thr172-AMPKα in shGTPBP3-1, shGTPBP3-2 and negative control (NC) cells treated (+) or not (-) with AMPKα activator AICAR (1 mM) for 1 h or with AMPKα inhibitor Compound C (5 μM) for 1 h. The filter was also probed with AMPKα. **(B)** qRT-PCR analysis of *UCP2* mRNA expression in shGTPBP3-1, shGTPBP3-2 and NC cells treated or not with AICAR (1 mM) for 1 and 48 h or with Compound C (CC, 5 μM) for 1 h. **(C)** qRT-PCR analysis of *MPC1* mRNA expression in shGTPBP3-1, shGTPBP3-2 and NC cells treated or not with AICAR (1 mM) or with Compound C (CC, 5 μM), both for 1 h. All data are the mean ± SEM of at least three independent biological replicates. Differences were found to be statistically significant at *p<0.05, **p<0.01, ***p<0.001. n.s.: non-significant differences, A.U.: arbitrary units.

Activation of AMPK has been involved in the up-regulation of UCP2 expression [71–73]. Therefore, we analyzed the relationships between AMPK activation and *UCP2* mRNA induction in shGTPBP3 and NC cells. We found that treatment with AICAR (1 mM) for 1 h, which led to AMPK activation (Fig 9A), increased the UCP2 mRNA levels in NC cells, but not in shGTPBP3 cells (Fig 9B). It is possible that either exacerbated AMPK activation in shGTPBP3 cells or the activation of AMPK-independent pathways by AICAR [74] interfered in some way with UCP2 induction in our cell lines. In fact, a longer treatment with AICAR (1 mM, 48 h), which also increased AMPK phosphorylation (see Fig 8A), did not affect the UCP2 levels in NC cells and reduced the UCP2 expression in shGTPBP3 cells to similar levels as NC cells (Fig 9B). Notably, treatment with the AMPK inhibitor compound C significantly decreased the UCP2 mRNA levels in shGTPBP3 cells (Fig 9B), suggesting that the UCP2 induction observed in these cells is mediated by AMPK.

It has been recently proposed that UCP2 is a metabolite transporter that, by exporting malate, oxalacetate and aspartate out of mitochondria, limits mitochondrial catabolism of pyruvate originating from glucose, while promoting the oxidation of alternative substrates such as glutamine and fatty acids [44, 49]. According to this idea, UCP2 induction in shGTPBP3 cells might be a compensatory mechanism for the Complex I defect exhibited by these cells, favoring the electron flow from Complex II and ETFs.

Pyruvate is a key molecule that lies at the intersection of multiple pathways, including glycolysis and TCAC; as such, its transport into the mitochondrial matrix, which is facilitated by the mitochondrial pyruvate carrier (MPC), influences ATP production by oxidative phosphorylation and multiple metabolic pathways connected with the TCAC. Thus aberrant pyruvate metabolism plays a prominent role in numerous diseases, including cardiac failure [75]. It has been shown that the suppression of pyruvate transport by a transcriptional or pharmacological inhibition of the MPC induces a form of metabolic flexibility associated with the use of lipids and glutamine to maintain the tricarboxylic acid cycle [76, 77], which is reminiscent to the metabolic shift promoted by UCP2 [44, 47, 49]. These data, together with the fact that *GTPBP3* patients usually present hypertrophic cardiomyopathy and lactic acidosis [28], led us to hypothesize that the down-regulation of MPC could be part of an adaptive response of the GTPBP3-defective cells. Thus we analyzed the expression of MPC1, one of the two paralogous subunits that form the human MPC [78], in shGTPBP3 cells and found a decrease of about 40–50% in both the mRNA and protein levels (Fig 9C and S3 Fig). Then, we wondered whether AMPK activation could play some role in this effect. We found that incubation with AICAR for 1 h markedly lowered the expression of *MPC1* in NC cells (50%), but had no additional effect in shGTPBP3 cells (Fig 9C). Notably, treatment with the AMPK inhibitor compound C significantly increased the *MPC1* mRNA levels in shGTPBP3 cells to those observed in untreated NC cells (Fig 9C). This finding suggests that *MPC1* expression is down-regulated by AMPK activation in shGTPBP3 cells.

As far as we know, the data presented here provide the first evidence of a link between AMPK activation and regulation of MPC and Complex I assembly factors.

### Expression of genes involved in glycolysis and fatty acid oxidation is induced in shGTPBP3 cells

**Fig 10:**
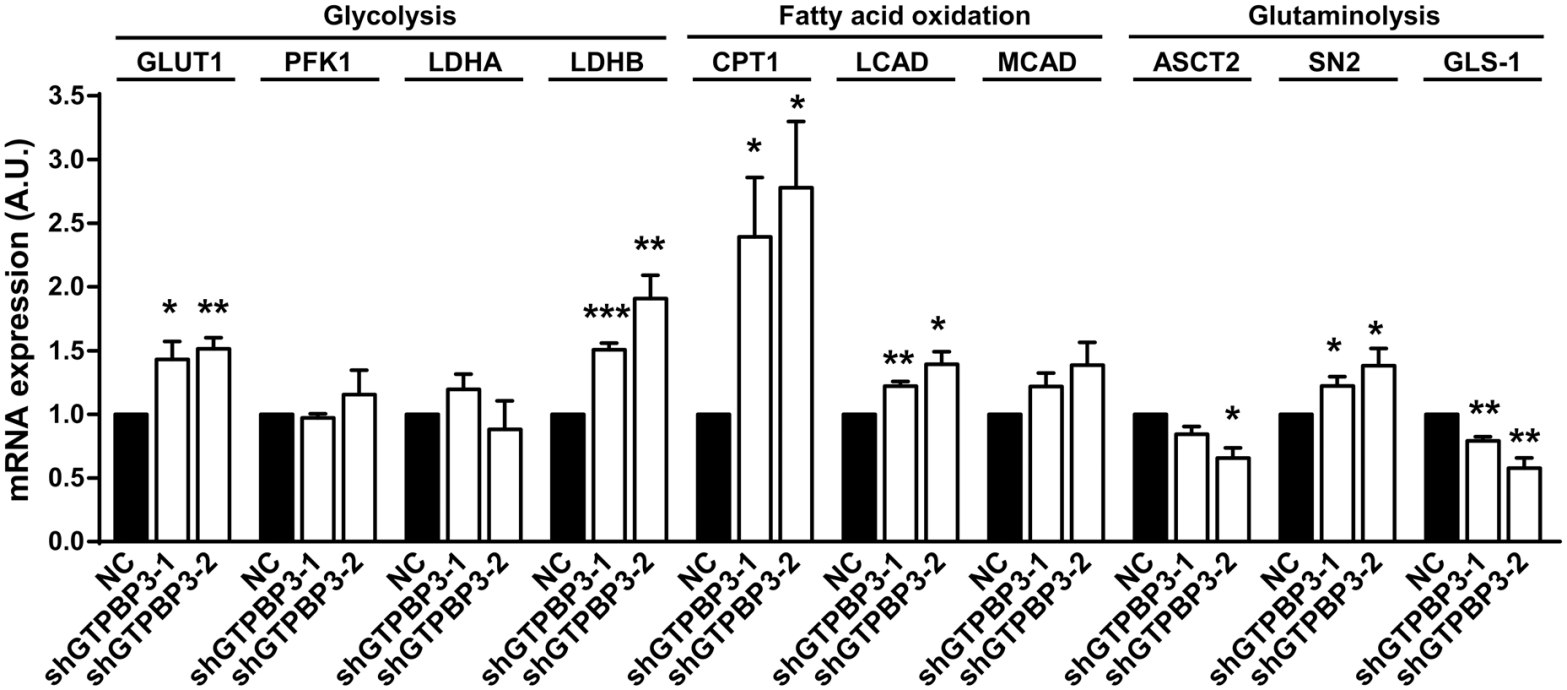
Increased mRNA expression in shGTPBP3 cells of genes involved in glycolysis and fatty acid oxidation. qRT-PCR analysis of mRNA expression of genes related to: 1) glycolysis (*GLUT1*: glucose transporter 1, *PKF1*: phosphofructokinase, and *LDHA* and *LDHB*: lactate dehydrogenase A and B, respectively), 2) fatty acid oxidation (*CPT1*: carnitine palmitoyltransferase I, *LCAD*: long-chain acyl-CoA dehydrogenase, and *MCAD*: medium-chain acyl-CoA dehydrogenase), and 3) glutaminolysis (*ASCT2*: glutamine/amino acid transporter 2, *SN2*: glutamine/amino acid transporter system N, and *GLS*: glutaminase) in shGTPBP3-1, shGTPBP3-2 and NC cells. Data are the mean ± SEM of at least three independent biological replicates. Differences from NC values were found to be statistically significant at *p<0.05, p<0.01 and ***p<0.001. A.U.: arbitrary units.

Activation of AMPK mobilizes glucose into ATP-generating processes, including glycolysis and fatty acid oxidation [51, 52, 79]. Moreover, both the up-regulation of UCP2 as well as the down-regulation of MPC have been associated with a metabolic shift from pyruvate to glutamine and fatty acid oxidation as a means to provide reducing equivalents to the OXPHOS system [42, 44, 46, 47, 76]. Therefore, we examined the expression of genes involved in these pathways (glycolysis, fatty acid oxidation, and glutamine oxidation) to gain further insight into the metabolic changes that occur upon stable GTPBP3 depletion. We found that the mRNA levels of GLUT1, a glucose transporter that has been shown to be transcriptionally and post-translationally activated via AMPK [80, 81], and lactate dehydrogenase B (LDHB, the main isoenzyme in the heart that catalyzes the interconversion of pyruvate and lactate with the concomitant interconversion of NADH and NAD^+^) significantly increased in shGTPBP3 cells (Fig 10). These data suggest that glycolysis and production of lactate are activated in shGTPBP3 cells. No changes were observed in lactate dehydrogenase A, the major isoenzyme in liver and skeletal muscle, and phosphofructokinase (PFK1), the first regulatory site that commits glucose to catabolism by glycolysis (Fig 10). Notwithstanding, PFK1 is known to be indirectly activated by AMPK through phosphorylation of phosphofructokinase 2 (PFK2), which converts fructose 6 phosphate into fructose-2,6-bisphosphate, an allosteric stimulator of PFK1 [82]. Therefore, the possibility that PFK1 becomes activated in shGTPBP3 cells as an indirect consequence of AMPK activation cannot be excluded.

With respect to fatty acid oxidation, we studied the expression of CPT1 (the enzyme that mediates the transport of long-chain fatty acids across the outer mitochondrial membrane, leading to increased fatty acid flux into the mitochondria for β-oxidation), and long-chain acyl-CoA dehydrogenase (LCAD) and medium-chain acyl-CoA dehydrogenase (MCAD), which catalyze the initial step of β-oxidation of long- and medium-chain acyl-CoA substrates, respectively, using FAD as a cofactor [75]. As shown in Fig 10, we found a 2-fold increase in the mRNA levels of CPT1 and a significant increase in the mRNA expression of LCAD. These data suggest that stable down-regulation of *GTPBP3* increases fatty acid oxidation.

Finally, we explored the expression of some genes related to glutaminolysis; specifically, the genes that encode glutaminase and glutamine transporters ASCT2 and SN2. We found that while mRNA expression of the transporters was uneven, the mRNA levels of glutaminase (GLS-1), the enzyme that initiates the conversion pathway of glutamine to α-ketoglutarate, were significantly lowered in shGTPBP3 cells (Fig 10), pointing to glutaminolysis does not increase in these cells.

Altogether, our findings suggest that the inhibition of pyruvate oxidation due to the up-regulation of UCP2 and the down-regulation of MPC can rewire cellular metabolism to stimulate fatty acid oxidation as the main pathway to supply reducing equivalents to the OXPHOS system in shGTPBP3 cells.

## Discussion

Mutations in *GTPBP3* cause hypertrophic cardiomyopathy, lactic acidosis and encephalopathy, and they have been associated with a defect in mitochondrial translation [28], although the pathomechanism remains unclear. Here we show that defective *GTPBP3* expression affects the modification status of mt-tRNAs and triggers AMPK signaling, which leads to adaptive/maladaptive responses via the induction of UCP2 and the down-regulation of MPC and Complex I assembly factors.

GTPBP3 is homologous of proteins MnmE and MSS1 of *E*. *coli* and yeast, respectively; hence it is thought to be directly involved in the post-transcriptional modification of human mt-tRNAs, although no experimental evidence in support of this prediction has been published so far [7, 9, 28]. Due to the difficulty of directly analyzing the presence of τm^5^ in human mt-tRNAs, here we adopted an indirect method based on the higher sensitivity of hypomodified tRNAs towards digestion by angiogenin. Our results revealed that the defective expression of GTPBP3 altered the composition of mt-tRNA^Lys^ and mt-tRNA^Leu(UUR)^ as they were found to be more sensitive to angiogenin digestion than mt-tRNAs obtained from the control cells (Fig 2A). This represents the first experimental evidence of a role for GTPBP3 in mt-tRNA modification. In addition, we used APM-northern blotting analysis to demonstrate that thiolation at position 2 of U34, which is catalyzed by TRMU [21, 24], was not affected by the GTPBP3 defect (Fig 2C). This finding supports the idea that TRMU functions independently of the τm group being present at position 5.

It is noteworthy that the stable knocking down of *GTPBP3* expression left a remnant (about 25%) of the GTPBP3 protein (Fig 1B and 1C) and, accordingly, we assumed that a fraction of substrate tRNAs may contain a modified U34. Notwithstanding, the angiogenin assay showed a differential digestion pattern between the mt-tRNAs purified from shGTPBP3 and NC cells (Fig 2A). Therefore, we believe that this assay may be a useful qualitative approach to estimate the impairment of mt-tRNA modification in patient cells, even for the case of partial loss-of-function mutations.

tRNA modifications jointly introduced by the GTPBP3- and MTO1-family proteins are important for translational fidelity and an optimal translation rate [83–88]. Therefore, GTPBP3 defects are expected to result in some mitochondrial translation impairment. Indeed, a reduction in mitochondrial translation has been reported from the transient knocking down of *GTPBP3* with siRNAs and in cells from some, but not all, of the patients carrying *GTPBP3* mutations [28, 38]. In this work, we found that the stable knocking down of GTPBP3 did not cause consistent changes in overall protein synthesis, but a specific decrease in the labeling of Complex I subunits ND1 and ND3 was found to be significant (Fig 6B). Compensatory mechanisms might contribute to improve mitochondrial translation efficiency in shGTPBP3 cells. Nonetheless, the greater sensitivity of these cells to chloramphenicol (Fig 6C) suggests that mitochondrial translation could still be affected to some extent. The point is then why the apparently mild affectation of this process in shGTPBP3 cells resulted in such a marked drop in the levels and activity of Complex I and, in contrast, did not affect the levels and activity of Complex IV, which also includes mtDNA-encoded subunits (Figs 4B and 7D).

It is possible that translation of ND1 and ND3 mRNAs is especially dependent on properly modified mt-tRNAs and, in this manner, become more affected by the GTPBP3 defect. Structural and *in vivo* data support the notion that the xm^5^U-type modifications (where xm^5^U stands for diverse 5-methyluridine derivatives including carboxymethyluridine, methylaminomethyluridine and taurinomethyluridine) are important for modulating the geometry of the codon:anticodon pairs at the wobble position and, thus, the relative efficiency of anticodons in reading cognate codons [85, 89, 90]. Lack of xm^5^U-type modifications causes translational frameshifting *in vivo* [83, 84], which may be favored by the concurrence of several factors, including translational pausing and the presence of sequences in the mRNA predisposing to slippage [83, 84, 91], which are poorly defined in human mt-mRNAs [92]. Accordingly, we speculate that mt-tRNA hypomodification due to the down-regulation of GTPBP3 affects the translation of ND1 and ND3 to a greater extent, thus leading to a certain impairment of Complex I, an alteration of the AMP/ATP ratio, the activation of AMPK-dependent retrograde signaling pathways and, finally, to down-regulation of the assembly factors NDUFAF3 and NDUFAF4, which, in turn, aggravates Complex I dysfunction. Another, not necessarily alternative, possibility is that mt-tRNA hypomodification promotes misincorporation of amino acids during translation so that qualitative alterations of mtDNA-encoded subunits (mainly of Complex I subunits) play a pathogenic role in shGTPBP3 cells. Structural data indicate that the xm^5^U-type modifications are not required to prevent the codon-anticodon U3•U34 wobbling when the tRNA anticodon is pyrimidine-rich, as occurs in tRNAs decoding Lys, but could be necessary in other cases [89]. Accordingly, loss of the U34 modification could promote the mispairing of certain hypomodified mt-tRNAs with near-cognate codons. Misincorporation may also be determined by tRNA competition between cognate and near-cognate tRNAs as higher error frequencies result from lower competition from low-abundance tRNAs [93]. The relative abundance of mt-tRNAs could be modulated, for instance, by the particular stability of each hypomodified mt-tRNA species.

Other mechanisms might also contribute to the phenotype of shGTPBP3 cells. Thus hypomodified mt-tRNAs might directly perform a signaling function and participate in mitochondria-nucleus cross-talk. This hypothesis is based on the finding that loss of the wobble uridine modification in yeast cytosolic tRNAs has been found to affect gene expression by perturbing cell signaling in a translation-independent manner [94]. Moreover, some reports describe that mt-tRNAs may be exported to the cytoplasm and subsequently associate with argonauta (AGO) proteins, suggesting a role for mt-tRNAs in gene silencing [95, 96]. Whether this process is favored by mt-tRNA hypomodification remains to be explored. A different possibility is that GTPBP3 has an additional function to mt-tRNA modification, which may also contribute to the functional state of mitochondria. Particularly interesting is the recent finding that its partner protein MTO1 interacts with mitoribosomal proteins in an RNA-independent manner, which suggests that MTO1 helps in the assembly or stability of mitoribosomes [27].

*GTPBP3* and *MTO1* mutations are associated with combined Complex I and IV deficiency, although affectation of these respiratory complexes is dependent on the cell type [25–27]. In our cell model of the *GTPBP3* defect (HEK293-derivative cells), we found only a consistent affectation of Complex I (Figs 4B and 7D). Nonetheless, it is possible that a greater inactivation of the GTPBP3 function could lead to the impairment of Complex IV in this cell type since we have occasionally observed a drop in the steady-state levels of the COX IV subunit (Fig 7B).

AMPK is activated in shGTPBP3 cells, which is probably the result of the relatively low ATP levels (Fig 4A and 4D). It is known that AMPK activation promotes the inhibition of energy-consuming biosynthesis pathways and stimulates ATP-producing catabolic pathways, like glycolysis and fatty acid oxidation, but AMPK activation also participates in the regulation of many other functions, including regulation of mitochondrial biogenesis and disposal, autophagy and cell growth [51, 52, 54, 61, 79]. Therefore in shGTPBP3 cells, AMPK activation could be responsible for the observed reduced cell growth (Fig 5A and 5B), activation of autophagy (Fig 5C and 5D) and the maintenance of the relative mtDNA copy number (Fig 5I).

**Fig 11:**
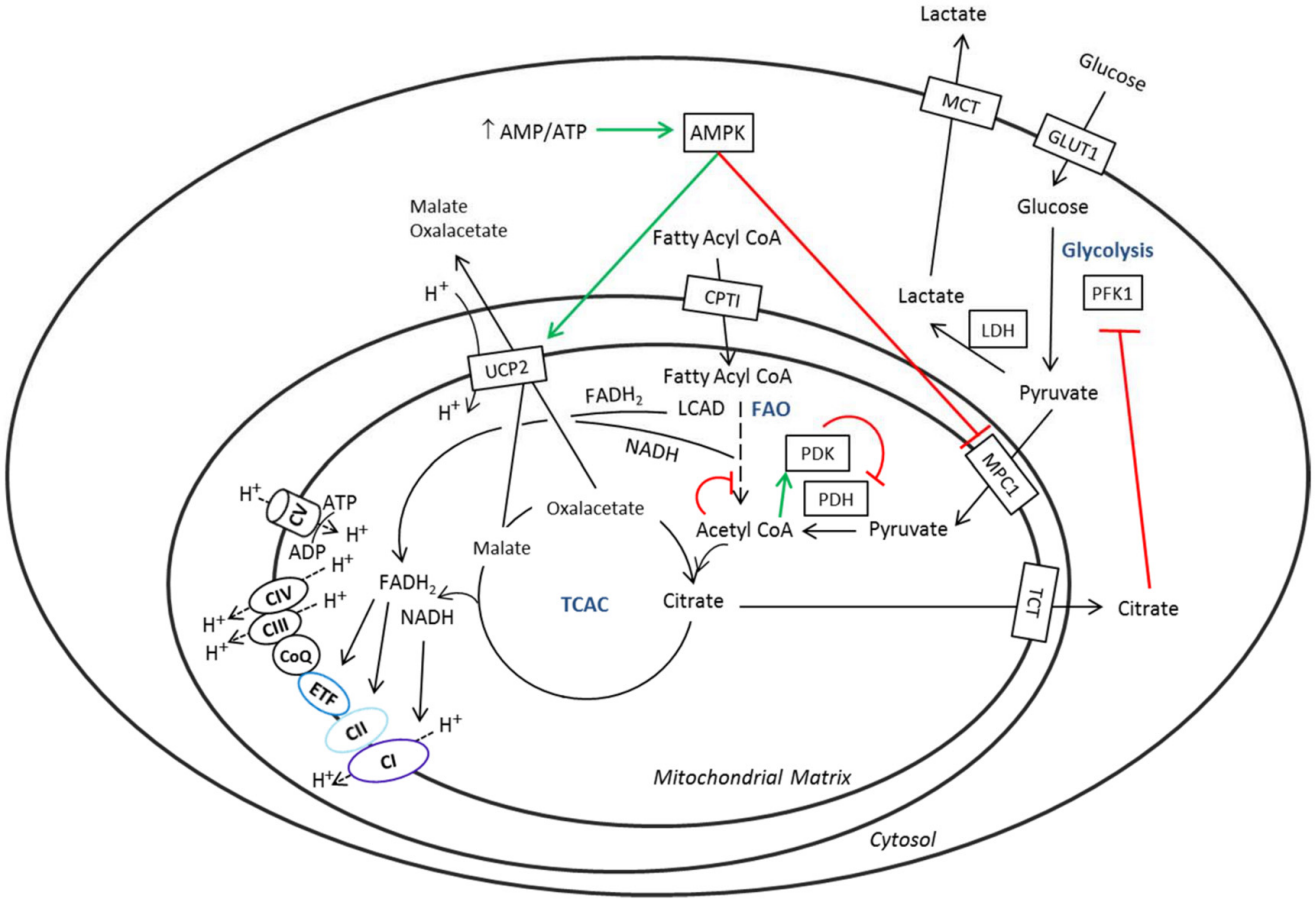
Proposed scheme of glucose and fatty acid metabolic pathways in shGTPBP3 cells. Glucose uptake is regulated by the translocation of the glucose transporters (e.g., GLUT1) to the cell membrane. In glycolysis, the first regulatory step that commits glucose to catabolism is controlled by phosphofructokinase (PFK1), whereas the last step is catalyzed by pyruvate kinase to produce pyruvate and ATP. Pyruvate is either oxidized (glucose oxidation) or converted to lactate by lactate dehydrogenase (LDH). When glycolysis is coupled to glucose oxidation, pyruvate enters the mitochondria by means of the mitochondrial pyruvate carrier (MPC) and is converted into acetyl CoA by pyruvate dehydrogenase (PDH), which is regulated by the action of pyruvate kinase (PDK) and pyruvate phosphatase. Finally, acetyl CoA incorporates into the tricarboxylic acid cycle (TCAC). NADH and FADH_2_ generated by TCAC enzymes are funneled into the mitochondrial electron transport chain through Complex I and Complex II, respectively. Long chain fatty acids use the carnitine shuttle, which includes carnitine palmitoyltransferase I (CPTI), to cross the mitochondrial membranes. Fatty acid oxidation (FAO) produces acetyl-CoA, which incorporates to the TCAC, and NADH and FADH_2_, which are used in the electron transport chain via Complex I and ETF-ubiquinone oxidoreductase, respectively. Fatty acid and glucose metabolism can regulate each other by the Randle Cycle [75, 117]. In shGTPBP3 cells, where Complex I is impaired, AMPK activation results in down-regulation of MPC1 (one of the two subunits that form MPC) and up-regulation of UCP2. The increase of UCP2 in shGTPBP3 cells could favor the export of malate and oxaloacetate (OAA) out of mitochondria and promote FAO. The decrease of MPC may also be part of an adaptive response that invokes FAO and oxidation of branched-chain amino acids to provide reducing equivalents to the OXPHOS system. However, insufficient recycling of NADH to NAD^+^, due to the low activity of Complex I, may limit the effects of the metabolic reprogramming on the OXPHOS capacity. This situation, together with a reverse operation of Complex V aimed to prevent a drastic drop of the membrane potential, could explain the low ATP levels in shGTPBP3 cells. These reduced ATP levels and the uncoupling of glycolysis from pyruvate oxidation, which can l increase proton and lactate production could be detrimental for tissues like the heart. In the figure, red and green lines indicate regulatory actions.

AMPK activation also appears to be related to the *UCP2* induction and the down-regulation of *NDUFAF3*, *NDUFAF4*, and *MPC1* expression in shGTPBP3 cells (Figs 3F, 3H, 7E and 9C; S3 Fig). It has been shown that UCP2 reconstituted in lipid vesicles catalyzes the exchange of malate, oxalacetate and aspartate for phosphate plus a proton from opposite sides of the membrane [49]. Thus it is thought that, by exporting C4 compounds out of mitochondria, UCP2 brakes the entry of glucose into the oxidation pathway while promoting oxidation of alternative substrates like fatty acids and glutamine [42, 44, 46, 47, 49]. Interestingly, we detected an increase of the *CPT1* and *LCAD* mRNA levels in shGTPBP3 cells (Fig 10), which points to increased fatty acid oxidation in these cells as a means to provide FADH_2_ to the respiratory chain via ETF-ubiquinone oxidoreductase (Fig 11). Given that UCP2 promotes the transport of C4 metabolites, including oxalacetate, out of mitochondria, and fatty acid oxidation produces acetyl-CoA but not oxalacetate, some anaplerotic reaction may act to replenish TCA cycle (TCAC) intermediates that have presumably been extracted by UCP2 in shGTPBP3 cells. The increased Complex II activity in these cells (Fig 4B) led us to suspect that glutaminolysis could play a role in maintaining homeostasis of TCAC by increasing the anaplerotic flux via glutamate and α-ketoglutarate, and, in this way, the levels of the Complex II substrate succinate. However, we found that the mRNA levels of glutaminase, the enzyme responsible for the transformation of glutamine into glutamate diminished in shGTPBP3 cells (Fig 10), suggesting that glutaminolysis is not stimulated in these cells.

The delivery of reducing equivalents to the OXPHOS system from ETF-ubiquinone oxidoreductase as a result of fatty acid oxidation (FAO) promoted by UCP2 may be a compensatory mechanism for the defect of Complex I in shGTPBP3 cells. The slightly better maintenance of oxygen consumption (Fig 3B) and lower ROS production (Fig 3C and 3D) compared to those observed in transiently knocked-down cells [38] would be in line with this hypothesis as: 1) there are data that document a physical association of FAO proteins with OXPHOS supercomplexes [97], which may improve electron transport efficiency and, therefore, respiration; 2) the oxidation of fatty acids, despite being a powerful source of FADH_2_, does not lead to high ROS generation [98]. Moreover, considering that the inhibition of the MPC activity has been shown to induce a reprogramming of mitochondrial metabolism to use lipids and amino acids as catabolic and anabolic fuels [76, 77], we think that the *MPC1* down-regulation in shGTPBP3 cells is part of an adaptive response that invokes the use of fatty acids and, perhaps, branched-chain amino acids to provide reduced equivalents to the respiratory chain. Branched-chain amino acids can be converted, after several reactions, either into acetyl-CoA or succinyl-CoA that enter the TCAC. The increased activity of Complex II observed in shGTPBP3 cells (Fig 4B) may be then aimed at maximally exploiting the existing succinate levels to fuel FADH_2_ reducing equivalents to the OXPHOS system.

It is striking that, despite the several mechanisms apparently evolved by shGTPBP3 cells to compensate the Complex I deficiency, their ATP levels remain low. Reasons for this can be, among others, a reverse operation of Complex V (Fig 4B), which could help to prevent a drastic drop of the membrane potential due to both Complex I deficiency and UCP2 activity, and an insufficient recycling of NADH to NAD^+^, due to the low Complex I activity, which may limit the β-oxidation of fatty acids and mitochondrial degradation of branched-chain amino acids [99, 100].

A consequence of the metabolic shift promoted by the increase of UCP2 and the decrease of MPC1 would be that despite glycolysis (from glucose to pyruvate) could take place inside shGTPBP3 cells, as suggested by our data (Fig 10), most of the pyruvate would not be used by mitochondria and would be converted into lactate by lactate dehydrogenase (Fig 11). This step is important because it converts cytosolic NADH back to NAD^+^ so that it is available for glycolysis to continue. The uncoupling between glycolysis and oxidative phosphorylation has been shown to increase proton and lactate production in the heart, which can potentially be detrimental to this organ [75]. It is possible that an uncoupling between glycolysis and oxidative phosphorylation may contribute to hypertrophic cardiomyopathy and lactic acidosis in GTPBP3 patients. Moreover, the reduced ATP levels exhibited by shGTPBP3 cells indicate that the compensatory response promoted by AMPK activation, up-regulation of UCP2 and down-regulation of MPC does not lead to a recovery of the ATP control levels, which may be detrimental for tissues with high energy demand like the heart [75].

In brief, we propose that the hypomodification of mt-tRNAs associated with GTPBP3 defects promotes a cell-type dependent activation of the AMPK signalling pathway, which leads to a metabolic shift from glucose to fatty acid oxidation via the up-regulation of UCP2 and the down-regulation of MPC. This response contributes to maintain ROS, oxygen consumption, and membrane-potential levels relatively close to the wild-type levels, although it can also lead to an altered regulation of fatty acid and glucose metabolism, which could be responsible for the high lactate levels and heart damage that occur in GTPBP3 patients.

## Materials and Methods

### Bacterial strains, plasmids and microbiological media

Two predesigned plasmids, each containing a specific shRNA against human GTPBP3, and a negative control shRNA plasmid (carrying a scrambled artificial sequence that did not match any human gene), were used for the stable knockdown of GTPBP3 (SABioscience SureSilencing shRNA plasmids catalog ID # KH17194H). *E*. *coli* DH5α cells were used for the overproduction and purification of shRNA plasmids. *E*. *coli* native tRNA^Lys^ was purified from strains BW25113 and BW25113 *mnmE*::*kan*. Bacterial strains were grown in LBT (Luria-Bertani-broth containing 40 μg/ml thymine). Antibiotics were added when required (ampicillin at 100 μg/ml; kanamycin at 80 μg/ml).

### Cell culture and plasmid transfections

Human HEK-293 cells (ATCC CRL 1573) were grown in full medium: Minimum Essential Medium (Sigma) supplemented with 10% heat-inactivated fetal bovine serum (Gibco) and 100 units/ml penicillin G (Sigma). This cell line was used for stable transfections. Cells were transfected with shRNA plasmids (final concentration: 0.5 μg/ml) using FuGene 6 transfection reagent (Roche) according to the manufacturer's specifications. Antibiotic selection (hygromycin B, 200 μg/ml) was started 24 h after transfection.

### RNA isolation and qRT-PCR

Total and small RNA were isolated using TRIzol reagent (Invitrogen) and NucleoSpin miRNA kit (Macherey-Nagel), respectively, following the manufacturer's instructions. To quantify mRNA levels, one-step qRT-PCRs were performed in an Applied Biosystems Step-One Real-Time PCR System. 50–300 ng of total RNA were reverse-transcribed and amplified by qPCR in 20 μl of total volume reaction containing specific primers (Sigma), Power SYBR Green PCR Master Mix, MultiScribe Reverse Transcriptase, and RNase Inhibitor (all from Applied Biosystems), according to the manufacturer’s instructions. Relative quantitation of mRNA levels was calculated using the comparative Ct method. *ACTB* gene was used as endogenous control. A list of the primers used in this work is provided in S1 Table.

### Isolation of native tRNA^Lys^ from *E*. *coli* and reverse-phase HPLC analysis of nucleosides

*E*.*coli* native tRNA^Lys^ molecules were purified from bulk tRNA by the Chaplet Column Chromatography method [101] using a biotinylated DNA probe complementary to a specific sequence of tRNA^Lys^ (S1 Table). Ethanol-precipitated tRNA was subsequently treated with nuclease P1 and *E*. *coli* alkaline phosphatase, and the resulting nucleosides were analyzed by high performance liquid chromatography (HPLC), as previously described [102]. HPLC analysis was monitored at 314 nm to achieve optimal absorption of thiolated nucleosides. Nucleosides were identified according to their UV spectra [103] and by comparison with appropriate controls.

### APM-Northern blotting analysis

The procedure was performed as previously described to assess the thiolation status of mitochondrial tRNAs [16]. Essentially, total RNA (7.5 μg) was run on a 15% polyacrylamide gel containing 7 M urea and 10 μg/ml APM and then transferred to positively charged nylon membranes (Roche). Pre-hybridization and hybridization steps were performed with Dig Easy Hyb (Roche) according to the manufacturer’s instructions. mt-tRNA^Lys^ was detected with a specific DIG-labeled synthetic oligodeoxynucleotide (S1 Table).

### In vitro cleavage reaction of human total small RNA and *E*. *coli* native tRNA^Lys^

For the cleavage reaction of human total small RNA, mixtures contained 1 μg of purified human total small RNA, 2.5 μg/ml recombinant angiogenin (rANG), 30 mM HEPES pH 7.4, 30 mM NaCl and 0.01% bovine serum albumin. Cleavage reactions of *E*. *coli* native tRNA^Lys^ contained 0.6 μg of purified native tRNA^Lys^, 12.5 μg/ml rANG, 30 mM HEPES pH 7.4, 30 mM NaCl, and 0.01% bovine serum albumin. Mixtures were incubated at 37°C for the indicated times and quenched by adding 5 μl of Gel Loading Buffer II (Life Technologies). Cleavage products from human and *E*. *coli* RNA samples were respectively resolved in 15% and 10% denaturating polyacrilamide gels with 7 M urea, and then transferred to positively charged nylon membranes (Mannheim Boehringer). Human mt-tRNA^Lys^, mt-tRNA^Leu(UUR)^ and mt-tRNA^Val^, and *E*. *coli* tRNA^Lys^ were detected with specific DIG-labeled synthetic oligodeoxynucleotides (see S1 Table).

### Measurement of intracellular ATP and oxygen consumption

The amount of ATP was measured with the ATP Bioluminescence Kit Assay Kit HSII (Roche), according to the manufacturer’s instructions. Luminescence was determined using the Spectra Max M5 (Molecular Devices). Endogenous respiration rate was measured as described previously [104]. Briefly, cells were collected by tripsinization and resuspended in culture media. Cells were transferred to a chamber equipped with a Clark-type oxygen electrode (YSI 5331A Oxygen Probe), pre-calibrated with air-saturated culture media, assuming 200 μM of O_2_ concentration. Measurements were collected using a data acquisition device (5300A monitor, YSI).

### Flow cytometry studies

Cells were detached at 37°C with trypsin-EDTA and resuspended in culture media. Mitochondrial membrane potential was measured by incubating cells in suspension (10^6^ cells/ml) with 100 nM MitoTracker Red CMXRos for 30 min at 37°C, and the emitted fluorescence (620 ± 20 nm band-pass filter) was recorded [105]. For ROS studies, cells were incubated for 30 min at 37°C with 5 μM hydroethidine or 5 μM dihydrorhodamine 123, washed twice with phosphate buffered saline (PBS), trypsinized, and the emitted fluorescences, red (filter as above) or green (525 ± 20 nm band-pass filter) for hydroethidine or dihydrorhodamine 123, respectively, were measured [106]. For cells subjected to extracellular oxidative stress, cells were incubated with 0.3 mM H_2_O_2_ for 2 h. After oxidant exposure, the medium was removed and intracellular H_2_O_2_ levels were detected as described above. For all the measurements, 10,000 cells were analysed and collected using a Cytomics FC 500 flow cytometer (Beckman Coulter).

### Antioxidant enzyme activities

To assay the activity of antioxidant enzymes, cells were collected by trypsinization and resuspended in PBS. Samples were homogenized by sonication, centrifuged for 10 min (12,000 g at 4°C) and supernatants were isolated. Catalase activity was estimated according to the spectrophotometric assay of Aebi [107], monitoring H_2_O_2_ consumption at 240 nm. SOD was measured spectrophotometrically at 550 nm by assessing the inhibition of cytochrome *c* reduction by O_2_^-^ generated by the xanthine/xanthine oxidase system [108]. Glutathione peroxidase (GSH-Px) was measured following the oxidation of NADPH by glutathione reductase monitored at 340 nm using cumene hydroperoxide as a substrate [109].

### Determination of activities of OXPHOS complexes

To assay the activity of OXPHOS complexes, we previously isolated an enriched mitochondrial fraction from negative control and shGTPBP3 cells by mechanical homogenization in hypotonic buffer as described [110]. The activities of the OXPHOS Complexes I, II, IV and V and of citrate synthase were measured in a Varian Cary 300 Conc spectrophotometer with a temperature controller. Essentialy, Complex I was measured by following the rate of NADH oxidation at 340 nm, incubating the mitochondrial homogenate at 30°C in 20 mM phosphate buffer (potassium phosphate, pH 8.0), 200 μM NADH, 0.1% bovine serum albumin (BSA)-EDTA, 1mM NaN_3_, 100 μM ubiquinone-1. To calculate the rotenone-sensitive rate of NADH oxidation, the inhibitor of Complex I rotenone was added to a final concentration of 5 μM [111]. Complex II was assessed by measuring the reduction of 2,6-dichlorophenolindophenol (DCPIP) at 600 nm in 50 mM Tris-phosphate buffer (pH 7.0), 1.5 mM potassium cyanide (KCN), 32 mM succinate and 100 mM DCPIP [111]. Complex IV activity was analyzed by monitoring the oxidation rate of reduced cytochrome *c* at 550 nm, incubating the mitochondrial homogenate at 25°C in 10 mM potassium phosphate buffer, pH 7.0, and 80 μM reduced cytochrome *c* [111]. Determination of the ATPase activity of Complex V was assayed by an ATPase coupled reaction, measuring the oxidation of NADH at 340 nm at 30°C in the presence and absence of oligomycin [112, 113]. The reaction mixture contained 50 mM HEPES-Mg buffer at pH 8.0, 0.2 mM NADH, 2.5 mM phosphoenolpyruvate, 5 μl of pyruvate-kinase (10 mg/ml) and 10 μl of lactate dehydrogenase (5 mg/ml) in the presence of 10 μl of antimycin A (0.2 mg/ml in 50% ethanol). The oligomycin-sensitive fraction was measured by adding 10 μl of oligomycin (0.2 mg/ml in 50% ethanol). Citrate synthase activity was determined by spectroscopy at 420 nm by incubating the mitochondrial homogenate at 30°C in 75 mM Tris-HCl, pH 8.0, 100 μM 5,5´-dithiobis-(2-nitrobenzoic) acid, 0.5 mM oxaloacetate, 350 μg/ml acetyl-coenzyme A and 0.1% Triton X-100 [111]. Specific activities were expressed as nmol x min^-1^ x mg protein^-1^, and referred to the specific activity of citrate synthase to correct for mitochondrial volume.

### Pulse-labeling of mitochondrial translation products

Labeling of mitochondrial translation products was performed using [^35^S]-methionine and [^35^S]-cysteine (EasyTag EXPRESS ^35^S Protein Labeling Mix, PerkinElmer) in intact cells for 1 h, as described previously [114]. Emetine (100 μg/ml) was used to inhibit cytoplasmic protein synthesis. Samples were loaded onto 15–20% polyacrylamide gradient gels and run at 10 mA for 16 h or until 1 h after the dye front ran out of gel [114]. Gels were fixed in a methanol-acetic acid solution, treated 30 min with Amplify (GE Healthcare), dried and exposed to Amersham Hyperfilm MP film with Hyperscreen intensifying screens (GE Healthcare) at -80°C for several days.

### Cell viability assays

Effects of chloramphenicol and cycloheximide inhibitors on the viability of cells was evaluated as described previously [67] using the CellTiter 96 AQueous One Solution Cell Proliferation Assay (Promega).

### Blue-Native PAGE and Western blot

BN-PAGE was performed similarly as described in detail elsewhere [24, 115]. Briefly, mitoplasts were prepared by treatment with 1.2 mg digitonin per mg of protein, and were then solubilized with 1% lauryl maltoside, which is a mild non-ionic detergent that promotes dissociation of the OXPHOS supercomplexes while complexes I-V are usually solubilized as individual membrane protein complexes. Samples containing 15 μg of protein were separated on 3–12% Bis-Tris Novex NativePAGE gel (Life Technologies). Estimation of the relative level of the assembled respiratory complexes I-IV was assessed by Western blot with commercially antibodies: mouse monoclonal anti-NDUFB8 antibody (sc-65237, Santa Cruz Biotechnology), mouse monoclonal anti-SDHA antibody (A11142, Molecular Probes), mouse monoclonal anti-Complex III subunit Core 1 antibody (459140, Invitrogen) and rabbit polyclonal anti-COX IV antibody (4850, Cell Signaling.). Complex V was detected with a rabbit polyclonal antibody [116].

For Western blot, cell extracts were prepared in lysis buffer (150 mM NaCl, 1% Nonidet P40, 0.5% sodium deoxycholate, 0.1% SDS and 50 mM Tris-HCl pH 8.0), containing 0.1 mM leupeptin and 1 mM phenylmethanesulphonyl fluoride. Proteins (100 μg) from the various lysates were separated by SDS/PAGE (10 or 15% acrylamide) and transferred to PVDF membranes (GE Healthcare, Amersham Biosciences). For immunodetection, the same aforementioned antibodies were used along with others: anti-GTPBP3 antibody purified from GTPBP3-His-inoculated rabbit serum [38], rabbit polyclonal anti-porin antibody (ab15895, Abcam), mouse monoclonal anti-ND1 antibody (H00004535-A01, Abnova), mouse monoclonal anti-NDUFS3 antibody (sc-58393, Santa Cruz Biotechnology), mouse monoclonal anti-COX II antibody (A6404, Molecular probes), rabbit monoclonal anti-phospho-AMPKα (Thr172) antibody (2535, Cell Signaling), rabbit polyclonal anti-AMPKα antibody (2532, Cell Signaling), rabbit polyclonal anti-LC3B antibody (3868, Cell Signaling), goat polyclonal anti-UCP2 (Santa Cruz Biotechnology, sc-6525), rabbit polyclonal anti-MPC1 (Abcam, ab74871) and rabbit polyclonal anti-actin antibody (A2066, Sigma). The anti-goat (A5420), anti-rabbit (A6154) and anti-mouse (A4416) IgG-horseradish peroxidase-conjugated secondary antibodies were obtained from Sigma. Protein bands were quantified by densitometric analysis with an Image Quant ECL (GE Healthcare).

### Mitochondrial DNA copy number quantification

Mitochondrial DNA quantification was performed by real-time PCR as previously described [67].

### Electron microscopy and morphometry

Cells were seeded on Lab-Tek chamber slides (Nunc), washed and fixed with 3% glutaraldehyde. Then, they were post-fixed in 1% osmium tetroxide for 1 h, rinsed, dehydrated, incubated for 2 h with 2% of uranyl acetate and embedded in Araldite (Sigma-Aldrich). Ultrathin sections were cut, stained with lead citrate, and examined under a Philips CM10 transmission electron microscope. For the morphometric study of each sample, 24 electron micrographs from two different experiments were randomly selected, which represented more than 500 μm^2^ cytoplasm area. The volume density of mitochondria was estimated by point counting, using a double-lattice test system with a 1.5 cm spacing. The volume density of mitochondria (Vv) was expressed as percent volume: Vv = (Pi/Pt) x 100 (%), where Pi is the number of points failing on each mitochondrial structure and Pt is the number of points failing on the cytoplasm. We estimated the mitochondrial number by counting mitochondrial profiles in the micrographs and referring to the area of cytoplasm. Mitochondrial size was determined by using the formula of the ellipse area = π x semi-major axis x semi-minor axis.

### Growth measurements

Cells were seeded at 1.5 x 10^5^ cells/well in five 6-well plates and were grown for one to five days in MEM medium supplemented with 10% heat-inactivated fetal bovine serum and 100 units/ml penicillin G. Cells corresponding to each day were detached with trypsin-EDTA, resuspended in culture media and proliferation was monitored by counting cell number using a Bürker chamber. The population doubling time in the exponential phase was obtained by exponential regression (http://www.doubling-time.com/compute.php).

### Autophagy analysis

To assess autophagy by LC3-II levels, cells were incubated for 1h under conditions of low (full medium) and high (phosphate buffered saline, PBS) proteolysis in the presence or not of lysosomal inhibitors (20 mM ClNH4 plus 0.1 mM leupeptin). Cell lysates were processed as described above for Western blot.

### Statistical analysis

Statistical analysis was performed using Student’s t test. The statistically significant differences between the means were indicated by asterisks (*p < 0.05, **p < 0.01 or ***p < 0.001), and non-significant differences by n.s.

## Supporting Information

S1 Fig *Escherichia coli* tRNA^Lys^ lacking the MnmE-dependent modification exhibits higher sensitivity to cleavage by angiogenin. **(A)** Northern analysis of *E*. *coli* tRNA^Lys^ purified from wild-type (wt) and *ΔmnmE* strains after *in vitro* angiogenin digestion for 1, 2 and 3 h. **(B)** HPLC analysis of *E*. *coli* native tRNA^Lys^ purified from the wild-type (wt) and *ΔmnmE* strains. Note that the final modification mnm^5^s^2^U was present only in the wild-type strain, whereas tRNA purified from the *mnmE* mutant strain carried s^2^U at position 34. Nucleoside s^4^U at position 8 was used as an internal control (its level did not change in both bacterial strains). Absorbance was monitored at 314 nm to maximize the detection of thiolated nucleosides.
(TIF)

S2 Fig qRT-PCR analysis of *NDUFS3* and *NDUFB8* mRNA expression in shGTPBP3-1, shGTPBP3-2 and negative control (NC) cells. Data are the mean ± SEM of at least three independent biological replicates. Differences from NC values were found to be statistically significant at *p<0.05 and **p<0.01. A.U.: arbitrary units.
(TIF)

S3 Fig (**A)** Western blot analysis of MPC1 in shGTPBP3-1, shGTPBP3-2 and NC cells. The filter was also probed with porin, as a loading control. **(B)** Densitometric analysis of MPC1 normalized to the loading control and represented as % of NC. Data are the mean ± SEM of at least three independent biological replicates. Differences from NC values were found to be statistically significant at *p<0.05 and **p<0.01.
(TIF)

S1 Table List of the oligonucleotide sequences used in this work. “Fw” indicates forward primer and “Rv” denotes reverse primer.
(DOCX)
