# Supplementary figures and images for: Defective Expression of the Mitochondrial-tRNA Modifying Enzyme GTPBP3 Triggers AMPK-Mediated Adaptive Responses Involving Complex I Assembly Factors, Uncoupling Protein 2, and the Mitochondrial Pyruvate Carrier
Source: PLoS One. 2015 Dec 7;10(12):e0144273. doi: 10.1371/journal.pone.0144273 (PMC4671719; doi:10.1371/journal.pone.0144273)

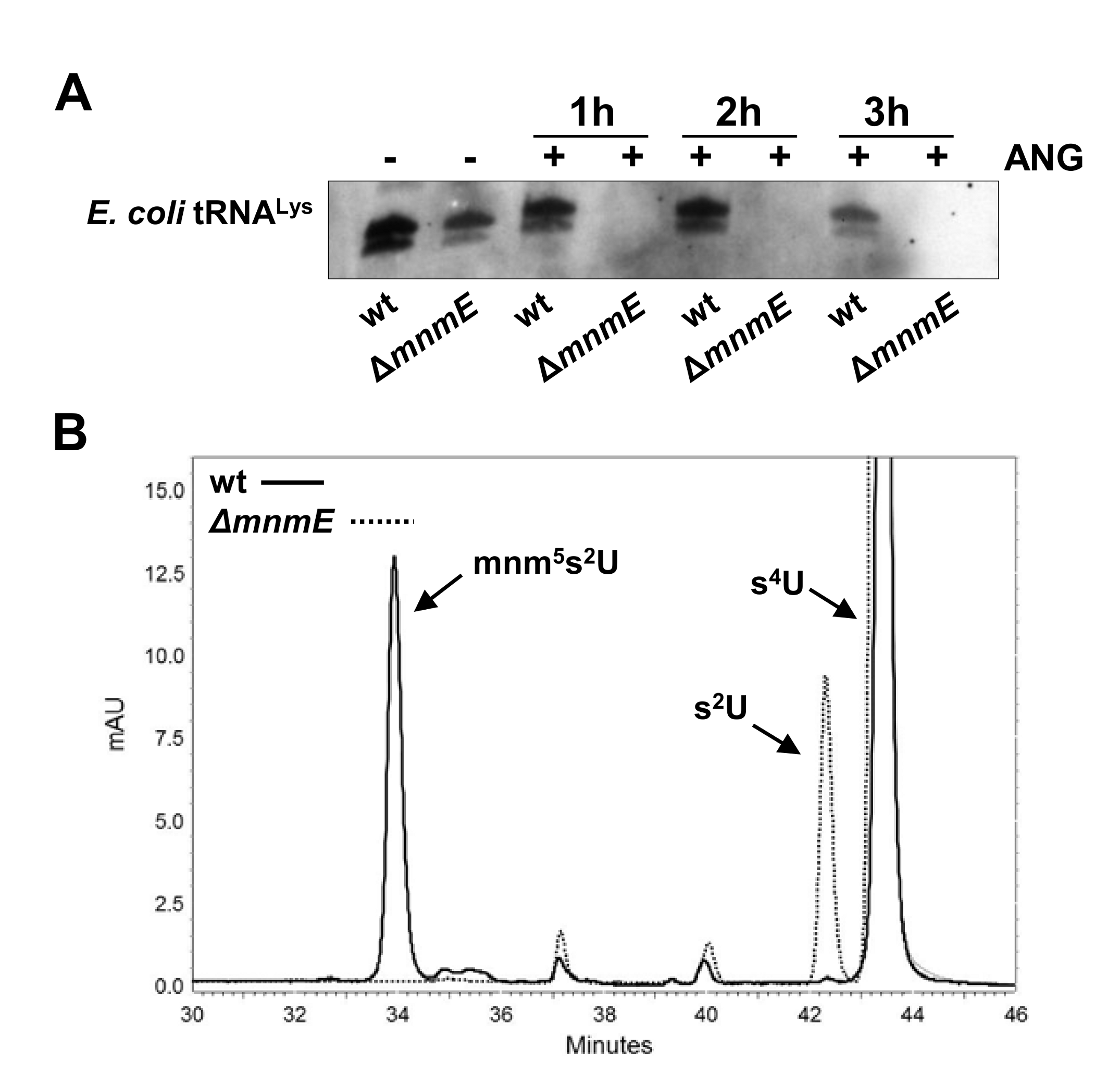

Supplement: S1 Fig — (A) Northern analysis of E. coli tRNALys purified from wild-type (wt) and ΔmnmE strains after in vitro angiogenin digestion for 1, 2 and 3 h. (B) HPLC analysis of E. coli native tRNALys purified from the wild-type (wt) and ΔmnmE strains. Note that the final modification mnm5s2U was present only in the wild-type strain, whereas tRNA purified from the mnmE mutant strain carried s2U at position 34. Nucleoside s4U at position 8 was used as an internal control (its level did not change in both bacterial strains). Absorbance was monitored at 314 nm to maximize the detection of thiolated nucleosides. (TIF) [file pone.0144273.s001.tif]

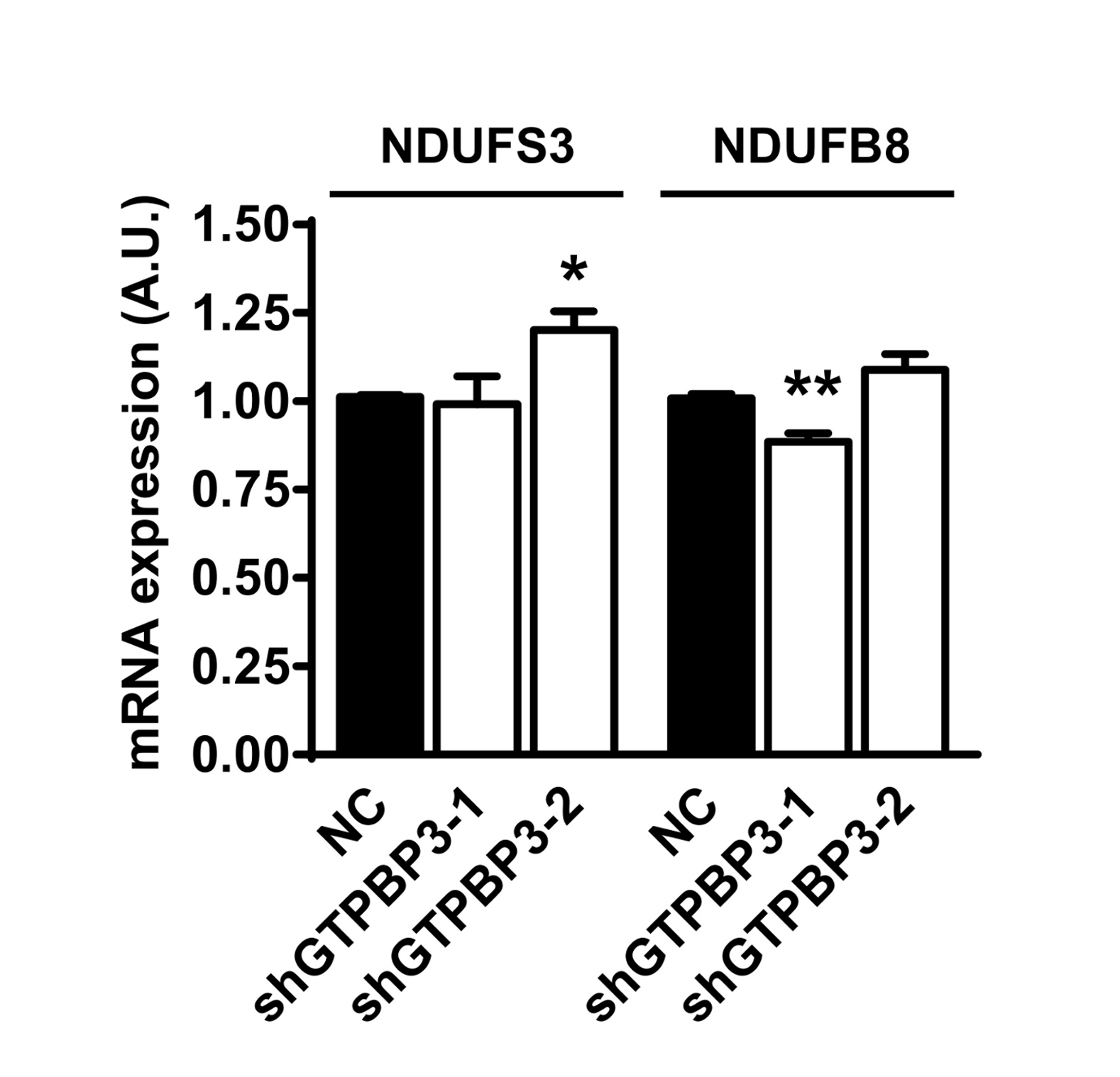

Supplement: S2 Fig — Data are the mean ± SEM of at least three independent biological replicates. Differences from NC values were found to be statistically significant at *p<0.05 and **p<0.01. A.U.: arbitrary units. (TIF) [file pone.0144273.s002.tif]

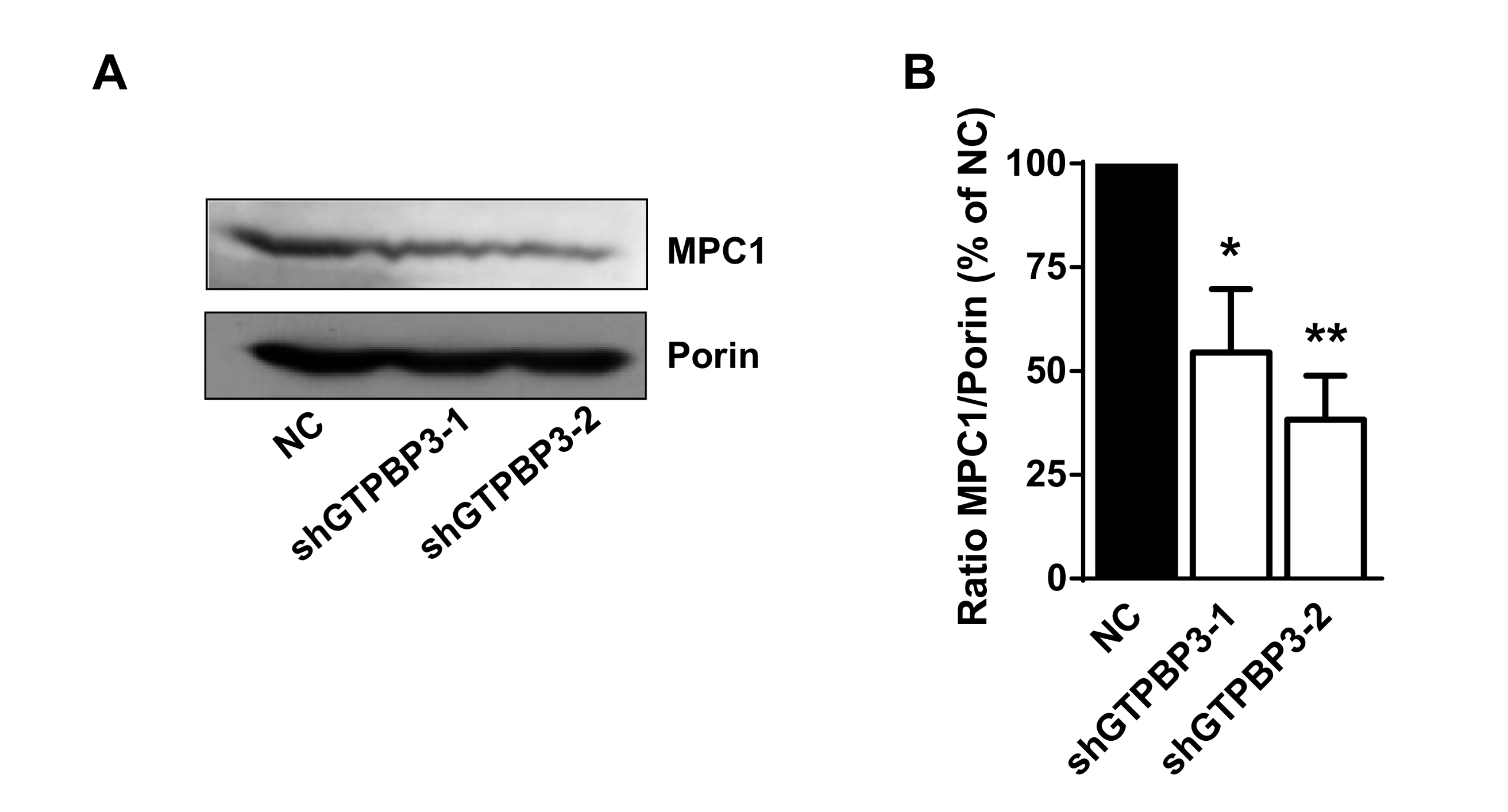

Supplement: S3 Fig — (A) Western blot analysis of MPC1 in shGTPBP3-1, shGTPBP3-2 and NC cells. The filter was also probed with porin, as a loading control. (B) Densitometric analysis of MPC1 normalized to the loading control and represented as % of NC. Data are the mean ± SEM of at least three independent biological replicates. Differences from NC values were found to be statistically significant at *p<0.05 and **p<0.01. (TIF) [file pone.0144273.s003.tif]
