# Supplementary material for: Defective Expression of the Mitochondrial-tRNA Modifying Enzyme GTPBP3 Triggers AMPK-Mediated Adaptive Responses Involving Complex I Assembly Factors, Uncoupling Protein 2, and the Mitochondrial Pyruvate Carrier
Source: PLoS One. 2015 Dec 7;10(12):e0144273. doi: 10.1371/journal.pone.0144273 (PMC4671719; doi:10.1371/journal.pone.0144273)
Supplement: S1 Table — “Fw” indicates forward primer and “Rv” denotes reverse primer. (DOCX) [file pone.0144273.s004.docx]

| **Gene name** | **Oligonucleotide name** | **Sequence (5'→3')** | **Assay** |
| --- | --- | --- | --- |
| **GTPBP3** | GTPBP3 Sab | GTPBP3 SaBioscience RT^2^qPCR primer assay | qRT-PCR |
|  | GTPBP3.3-Fw | GAGCTAGCTGCAGTGTGTGG | qRT-PCR |
|  | GTPBP3.3-Rv | GCCAGGTCTTTTGACTGCTT | qRT-PCR |
| **Actin** | Actin-Fw | TGAGCGCGGCTACAGCTT | qRT-PCR |
|  | Actin-Rv | TCCTTAATGTCACGCACGATT | qRT-PCR |
| **Thioxiredoxin-1** | Thioxiredoxin-1-Fw | GCCTTGCAAAATGATCAAGC | qRT-PCR |
|  | Thioxiredoxin-1-Rv | TTGGCTCCAGAAAATTCACC | qRT-PCR |
| **Thioxiredoxin-2** | Thioxiredoxin-2-Fw | CGCCATTGAGTATGAGGTGTCA | qRT-PCR |
|  | Thioxiredoxin-2-Rv | CCACCACGTCCCCATTCTT | qRT-PCR |
| **Peroxiredoxin-3** | Peroxiredoxin-3-Fw | GGCGTTCCAGTATGTAGAAACACA | qRT-PCR |
|  | Peroxiredoxin-3-Rv | GCTGGACTTGGCTTGATCGT | qRT-PCR |
| **Peroxiredoxin-5** | Peroxiredoxin-5-Fw | CAAGGCGGAAGGCAAGGT | qRT-PCR |
|  | Peroxiredoxin-5-Rv | CACCAGCGAATCATCTAGTAATAAGTCT | qRT-PCR |
| **UCP2** | UCP2-Fw | CATCGGCCTGTATGATTCTG | qRT-PCR |
|  | UCP2-Rv | TGGAATCGGACCTTTACCAC | qRT-PCR |
| **C20ORF7** | C20ORF7-Fw | GCCGACCAAATTTGACTACC | qRT-PCR |
|  | C20ORF7-Rv | GGGGGAAATTTCTGGGTATG | qRT-PCR |
| **NUBPL** | NUBPL-Fw | GGGTGGAGTCGGAAAATCTACTAC | qRT-PCR |
|  | NUBPL-Rv | TGGCCTTGGACGAATCGT | qRT-PCR |
| **NDUFAF3** | NDUFAF3-Fw | GGGCATTGCTGTGGAAGTG | qRT-PCR |
|  | NUDFAF3-Rv | GCCTTCATGACACAGGAAGTTG | qRT-PCR |
| **NDUFAF4** | NDUFAF4-Fw | CCTCTGTCGCTCCCAGACA | qRT-PCR |
|  | NDUFAF4-Rv | GCTTTTCATCTTTACGAGCAATCTC | qRT-PCR |
| **NDUFB8** | NDUFB8-Fw | CTACGAACCTTACCCGGATGAT | qRT-PCR |
|  | NDUFB8-Rv | CATGGATCTCTCTCATGCTGTGA | qRT-PCR |
| **NDUFS3** | NDUFS3-Fw | CACAAGCAGCTCTCAGCTTT | qRT-PCR |
|  | NDUFS3-Rv | TTGAAGCAGGACACCTGAAC | qRT-PCR |
| **MPC1** | MPC1-Fw | TGACATTCATGAGATTTGCCTACA | qRT-PCR |
|  | MPC1-Rv | TGAGCTGGGCTACTTCATTTGTT | qRT-PCR |
| **GLUT1** | GLUT1-Fw | CATCAACGCTGTCTTCTATTACTC | qRT-PCR |
|  | GLUT1-Rv | ATGCTCAGATAGGACATCCA | qRT-PCR |
| **PFK1** | PFK1.Fw | GCCGACTGGGTTTTTATTCCT | qRT-PCR |
|  | PFK1-Rv | ACGAGAACCACGGGTCCTT | qRT-PCR |
| **LDHA** | LDHA-Fw | GCCTGTATGGAGTGGAATGAA | qRT-PCR |
|  | LDHA-Rv | CCAGGATGTGTAGCCTTTGAG | qRT-PCR |
| **LDHB** | LDHB-Fw | GGGAAAGTCTCTGGCTGATGAA | qRT-PCR |
|  | LDHB-Rv | CTGTCACAGAGTAATCTTTATCGGC | qRT-PCR |
| **CPT1** | CPT1-Fw | TCCTTCCAACTCACATTCAG | qRT-PCR |
|  | CPT1-Rv | GGTGTCTGTCTCCTCTCC | qRT-PCR |
| **LCAD** | LCAD-Fw | AAGTGATGTTGTGATTGTAGTTG | qRT-PCR |
|  | LCAD-Rv | GAATAGTTCTGCGGTATCCTG | qRT-PCR |
| **MCAD** | MCAD-Fw | AATTAGTGAAGAATTGGCTTATGG | qRT-PCR |
|  | MCAD-Rv | ACATCAATGGCTCCTCAGTC | qRT-PCR |
| **ASCT2** | ASCT2-Fw | GAGGAATATCACCGGAACCA | qRT-PCR |
|  | ASCT2-Rv | AGGATGTTCATCCCCTCCA | qRT-PCR |
| **SN2** | SN2-Fw | GAGTTGCGGCCACTTCAG | qRT-PCR |
|  | SN2-Rv | TCCATTCATCTTTGGATCCTG | qRT-PCR |
| **GLS-1** | Glutaminase-Fw | GCATACACTGGAGATGTGTCTGC | qRT-PCR |
|  | Glutaminase-Rv | TGTCCAAAGTGTAGTGCTTCATCC | qRT-PCR |
| **COX II** | COX II-Fw | CGATCCCTCCCTTACCATCA | qPCR |
|  | COXII-Rv | CCGTAGTCGGTGTACTCGTAGGT | qPCR |
| **SDHA** | SDHA-Fw | TCTCCAGTGGCCAACAGTGTT | qPCR |
|  | SDHA-Rv | GCCCTCTTGTTCCCATCAAC | qPCR |
| **mt-tRNA^Lys^** | mt-tRNA^Lys^ dig | TGGTCACTGTAAAGAGGTGTTGGT | Northern blot |
| **mt-tRNA^Leu(UUR)^** | mt-tRNA^Leu(UUR)^ dig | GGAATTGAACCTCTGACTGTAAAGTTTTAAG | Northern blot |
| **mt-tRNA^Val^** | mt-tRNA^Val^ dig | GAAATCTCCTAAGTGTAAGTTGGGTGCTTTG | Northern blot |
| **tRNA^Lys(UUU)^** | 5tRNA^Lys(UUU)^ coli-dig | ACCAACTGAGCTAACGACCC | Northern blot |
| **tRNA^Lys(UUU)^** | Biotin-tRNA^Lys(UUU)^ | Biotin-TGGTGGGTCGTGCAGGATTCGAACCTG | tRNA isolation |
